# The efficacy of anti‐proteolytic peptide R7I in intestinal inflammation, function, microbiota, and metabolites by multi‐omics analysis in murine bacterial enteritis

**DOI:** 10.1002/btm2.10446

**Published:** 2022-11-08

**Authors:** Taotao Sun, Xuesheng Liu, Yunzhe Su, Zihang Wang, Baojing Cheng, Na Dong, Jiajun Wang, Anshan Shan

**Affiliations:** ^1^ Laboratory of Molecular Nutrition and Immunity, the Institute of Animal Nutrition Northeast Agricultural University Harbin China

**Keywords:** anti‐proteolytic, inflammatory bowel disease, multiple omics, oral drug delivery, therapeutic peptides

## Abstract

Increased antibiotic resistance poses a major limitation in tackling inflammatory bowel disease and presents a large challenge for global health care. Antimicrobial peptides (AMPs) are a potential class of antimicrobial agents. Here, we have designed the potential oral route for antimicrobial peptide R7I with anti‐proteolytic properties to deal with bacterial enteritis in mice. The results revealed that R7I protected the liver and gut from damage caused by inflammation. RNA‐Seq analysis indicated that R7I promoted digestion and absorption in the small intestine by upregulating transmembrane transporter activity, lipid and small molecule metabolic processes and other pathways, in addition to upregulating hepatic steroid biosynthesis and fatty acid degradation. For the gut microbiota, *Clostridia* were significantly reduced in the R7I‐treated group, and *Odoribacteraceae*, an efficient isoalloLCA‐synthesizing strain, was the main dominant strain, protecting the gut from potential pathogens. In addition, we further discovered that R7I reduced the accumulation of negative organic acid metabolites. Overall, R7I exerted better therapeutic and immunomodulatory potential in the bacterial enteritis model, greatly reduced the risk of disease onset, and provided a reference for the in vivo application of antimicrobial peptides.

## 1 INTRODUCTION

Inflammatory bowel disease (IBD), a global health care problem, is a chronic recurrent gastrointestinal inflammatory disease of complex etiology, subdivided into two phenotypes: Crohn's disease (CD) and ulcerative colitis (UC).^1^, ^2^ Recently, IBD incidence and prevalence continue to increase worldwide, with an estimated approximately 1.5 million IBD people in North America.^3^ Numerous studies have determined that IBD mainly affects the distal ileum and colon and involves complex interactions between intestinal flora, host genetic and immune factors, and environmental stimuli, among which enteric bacteria are critical in the pathogenesis of inflammatory bowel disease, especially *Escherichia coli*.^4^ *Escherichia coli* can adhere to and invade intestinal epithelial cells (IECs), survive and replicate inside macrophages without inducing cell death, and induce a high production of pro‐inflammatory cytokines and chemokines, thereby impairing the intestinal mucosal barrier and promoting IBD development.^5^, ^6^, ^7^ Ana Nemec et al. found that nitric oxide (NO) production was investigated in the lungs, thoracic aorta, heart, liver, spleen, kidneys, and brain of mice inoculated orally with *Escherichia coli ATCC* 25922.^8^ The generalized increase in NO production in the short and long terms indicates a host response to *E. coli* administered by the oral route of infection. Given this, using antibiotics (i.e., norfloxacin, ciprofloxacin, ofloxacin, and fleroxacin) to control the proliferation of *Escherichia coli* in intestinal mucosa may be a potential therapeutic strategy for IBD.^9^, ^10^

However, numerous studies have recently proved that the extended use or misuse of antibiotics can cause a persistent expansion of antibiotic resistance genes in the human gut microbiota and disrupt the normal microbiota ecological structure and epithelial barrier function, causing serious secondary infection.^11^, ^12^ As highlighted in the last WHO global report, antimicrobial resistance has reached an extremely alarming level in various infection sources, including *E. coli*. Therefore, existing antibiotics have failed to satisfy clinical treatment needs, and there is an urgent need to develop novel antimicrobial agents against bacterial infection enteritis.

Antimicrobial peptides (AMPs), as an important component of the innate immune system, are expressed on the epithelial surface and in neutrophils in mammals, and have broad‐spectrum activity against bacteria, fungi, viruses, and so on. Consequently, AMPs have garnered immense attention as a potential class of peptide‐based antimicrobial agents for combating bacterial infections.^13^, ^14^ Indeed, peptides‐based drugs constitute the fastest growing market in the pharmaceutical industry. According to the report, the global peptide therapeutics market was valued at US$ 25.0 Bn in 2018 and is anticipated to expand at a CAGR of 7.9% from 2019 to 2027 (https://www.transparencymarketresearch.com/peptide-therapeutics-market.html). However, most peptide drugs, including AMPs, are difficult to administer orally due to an enzymatic barrier that limits systemic bioavailability, yet oral administration is the recommended technique for treating gastrointestinal inflammatory illnesses.^15^, ^16^, ^17^ Accordingly, improving AMPs' protease resistance was crucial to substituting antibiotics in IBD treatment.

**FIGURE 1:**
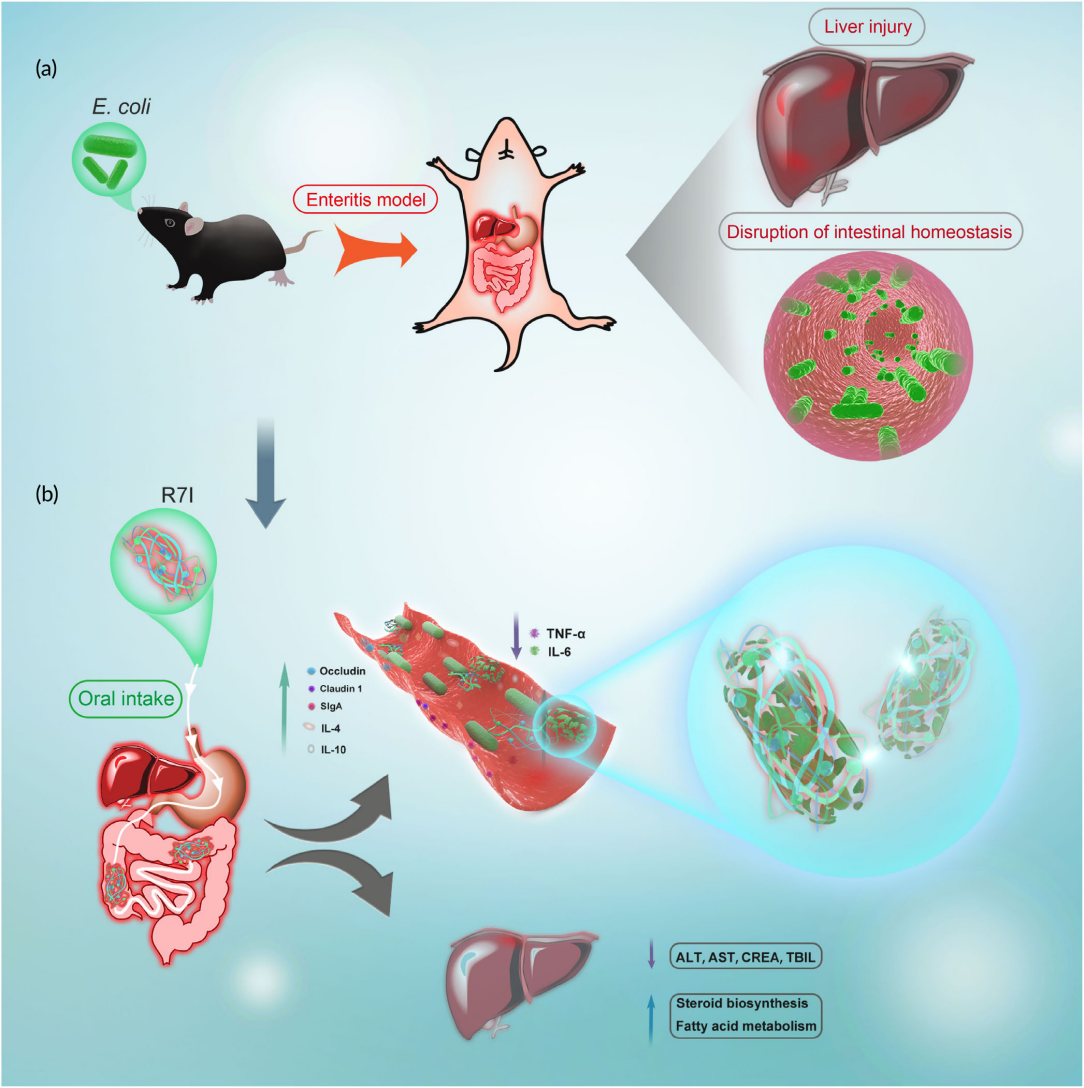
(a) Establishment of bacterial enteritis model in mice. The damage caused by *Escherichia coli* invading the mice's body through oral administration. (b) Therapeutic mechanism of anti‐proteolytic peptide R7I in mice.

In earlier research, we successfully developed an anti‐proteolytic peptide R7I (IRPI IRPI IRPI IRPI IRPI IRPI IRPI‐NH_2_), which had great bactericidal activity against all 16 tested Gram‐negative bacteria (GM_MBC_ = 4.97 μM) with low cytotoxicity. Additionally, R7I showed good salt tolerance and serum stability in vitro and still maintained excellent activity in a mouse peritonitis model. Importantly, compared with other anti‐proteolytic peptides containing unnatural amino acids, R7I was completely composed of natural amino acids and had dramatic resistance to a high concentration of protease hydrolysis (trypsin, chymotrypsin, and pepsin), suggesting that R7I could be produced cheaply through biological expression systems and had a broader application prospect.^18^ Therefore, we further investigated the efficacy of R7I in bacterial infection enteritis through oral administration. As displayed in Figure 1, we introduced an *E. coli*‐infected mouse model of enteritis to investigate the efficacy of R7I. Herein, we stated that R7I has excellent stability. In the inflammation model, R7I protected intestinal barrier and function, reducing intestinal inflammation and balancing intestinal flora disorders. In addition, R7I reduced liver damage and promoted liver‐related metabolic functions.

## 2 RESULTS

### 2.1 R7I had better stability and metabolic characteristics in vivo

**FIGURE 2:**
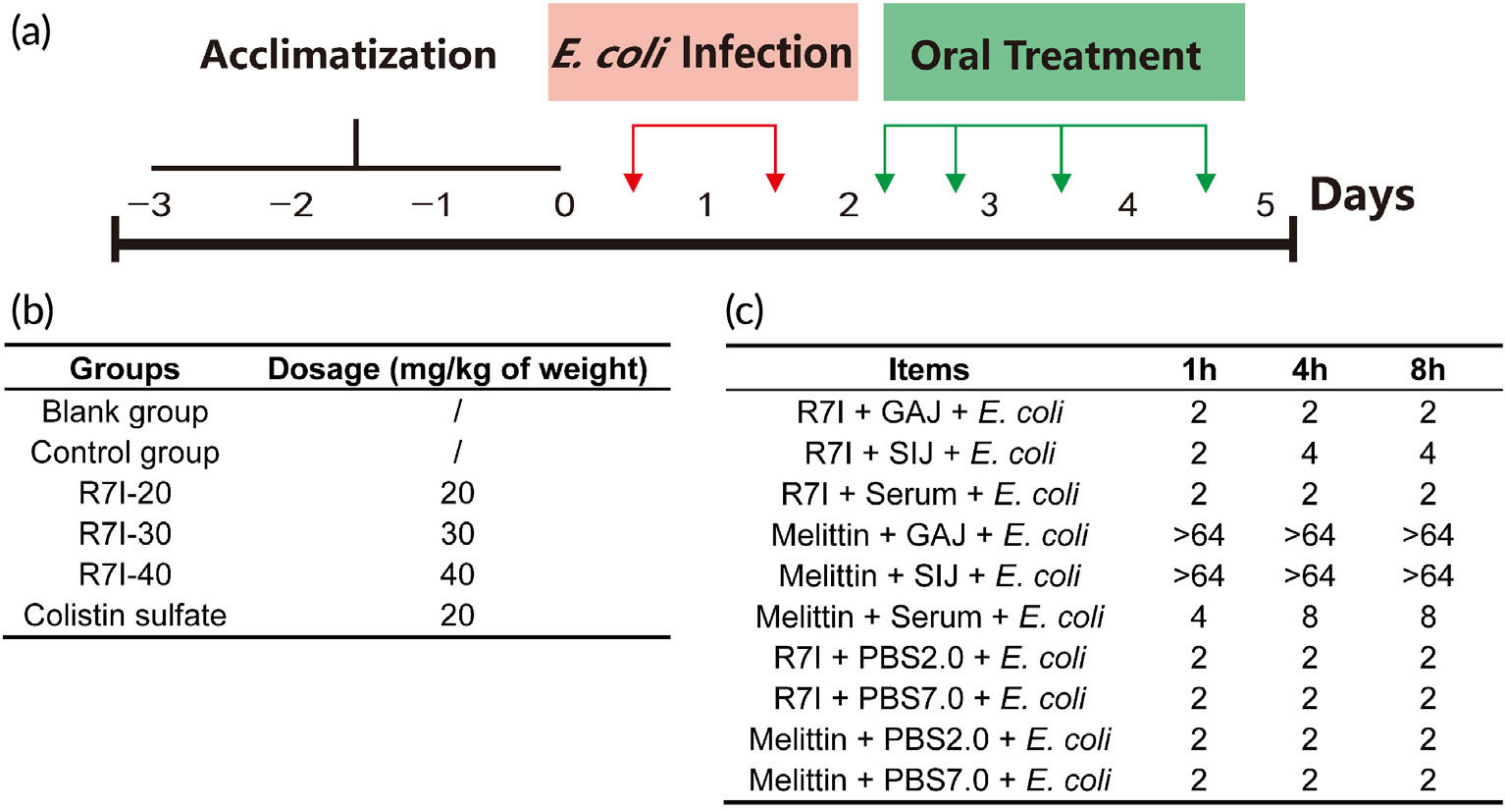
(a) Schematic diagram of the experimental scheme. Red and green arrows indicate the number of oral administrations and corresponding time points, respectively. (b) The experimental group design. (c) R7I MIC changes after incubation with serum, gastric juice, and small intestine fluid against *Escherichia coli* 25922. Minimum inhibitory concentrations (MICs, μM) were determined as the lowest peptide concentrations that killed greater than 95% of the bacterial cells. A value >64 indicates no detectable antibacterial activity at 64 μM. GAJ, gastric juice; SIJ, small intestinal juice; PBS2.0 and PBS7.0, phosphate‐buffered saline (pH = 2.0 or pH = 7.0); *E. coli*, *Escherichia coli* 25922

Previous studies have demonstrated that R7I exhibits excellent resistance to pepsin, trypsin, chymotrypsin, and proteinase K in vitro.^18^ To get closer to the real in vivo effect, we first extracted the serum, intestinal fluid, and gastric fluid from mice to determine the function of R7I in these fluids. As depicted in Figure 2c, we demonstrated that melittin as a control was completely inactivated in both gastric and small intestinal juices after incubation for 1 h. In contrast, the antimicrobial activity of R7I was unaffected after 8 h incubation in the gastric juice. In the small intestine fluid, MIC values against *E. coli* of R7I increased slightly from 2 to 4 μM after 4 h incubation, indicating that R7I activity was slightly affected in the small intestinal environment. Additionally, the serum environment had no negative effect on R7I activity, but slightly compromised the activity of melittin.

**FIGURE 3:**
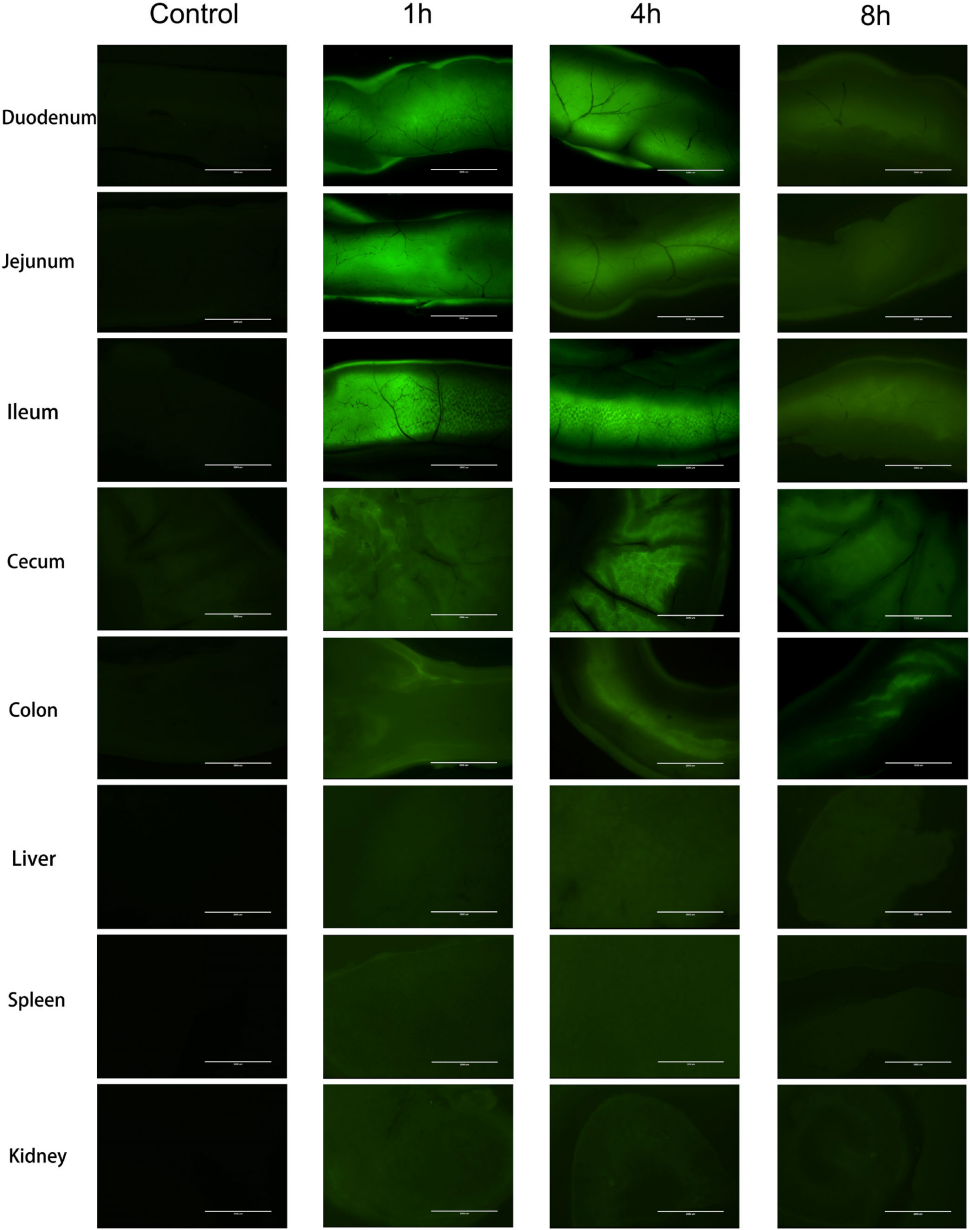
Fluorescence retention of anti‐enzymatic peptide R7I in mice. The C57BL/6 male mice were fasted for 12 h before treatment, after which the blank group was given saline and the experimental group was given 20 mg/kg R7I to start the clock. Samples were taken at 1 h, 4 h, and 8 h (organs and each mid‐gut), respectively. The blank group served as a control, and all samples were photographed using a fluorescent microscope (unified light source setting).

Subsequently, we observed the metabolic process of (FITC)‐labeled R7I in vivo. Figure 3 displays that R7I was present in large quantities in the small intestine after 1 h, and a high fluorescent signal remained in the intestine after 4 h. After 8 h, the fluorescent signal in the intestine and organs began to diminish.

Overall, the above data indicate that R7I had excellent resistance to enzymatic hydrolysis and had the potential for oral administration.

### 2.2 R7I could reduce inflammation in the liver and intestines

#### 2.2.1 R7I could protect intestinal mechanical and immune barriers

**FIGURE 4:**
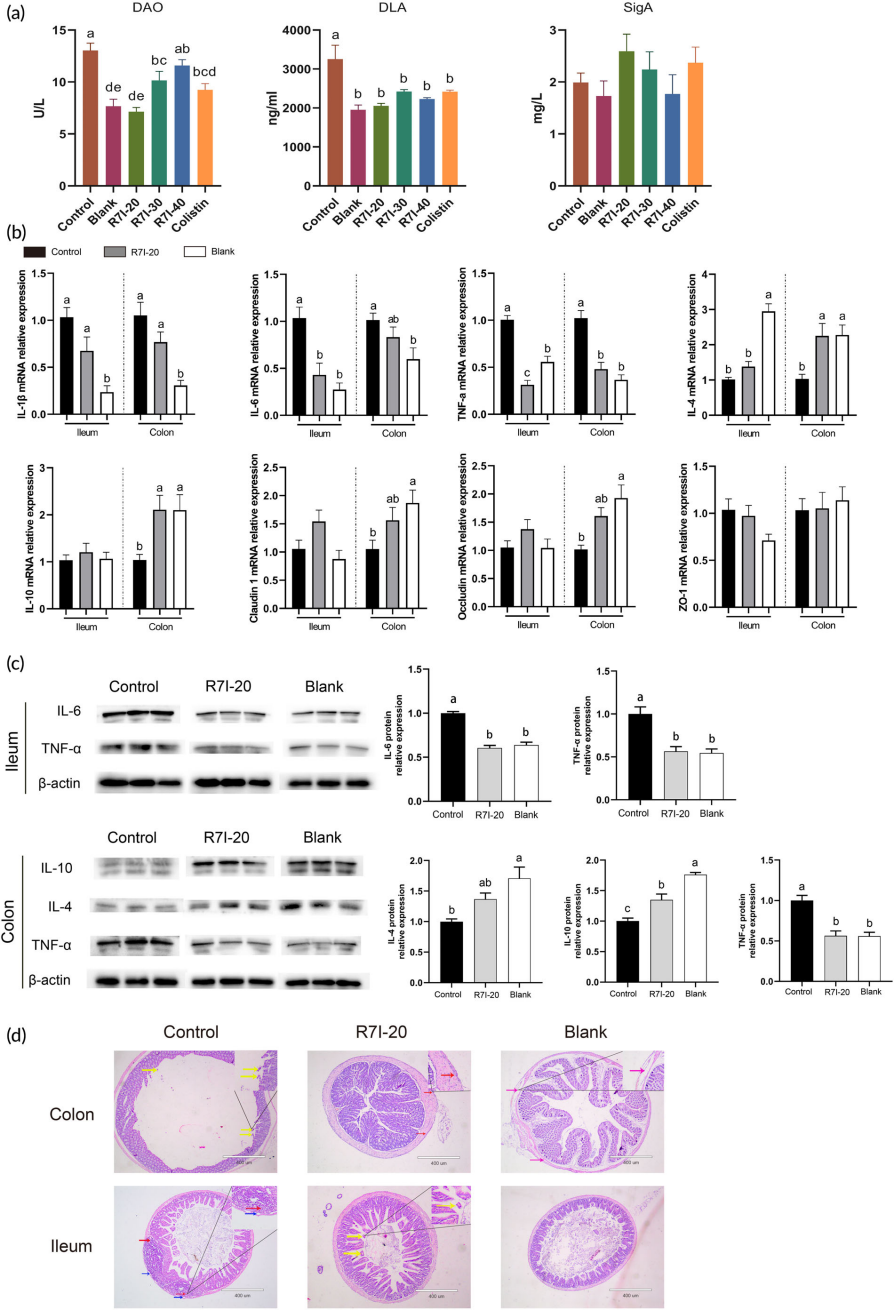
(a) Serum concentrations of d‐lactic acid, diamine oxidase, and secreted immunoglobulin A (SlgA) (*n* = 6). DLA, d‐lactic acid; DAO, diamine oxidase. (b) Inflammatory factor and tight junction proteins levels of relative mRNA expression in ileal and colonic tissues (*n* = 6). (c) Inflammatory factor proteins were examined by Western blot in the mouse colon and ileum (*n* = 3). A 40 μg sample of protein was taken. Beta‐actin was employed as an internal control. β‐Actin, beta‐actin, 42 kDa; IL‐4, interleukin 4, 30 kDa; IL‐6, interleukin 6, 24 kDa; IL‐10, interleukin 10, 19 kDa; TNF‐α, tumor necrosis factor alpha, 26 kDa. (d) H&E‐stained histological sections of intestinal tissues. Scale bars, 400 μm. In the ileum, red arrows stand for inflammatory cell infiltration; blue arrows stand for the partial separation of the mucosal layer from the lamina propria; yellow arrows stand for epithelial cell detachment. In the colon, yellow arrows stand for adhesion to intestinal villus structures and detachment of epithelial cells; red arrows stand for localized areas of cell proliferation in the serosal layer. The data were presented as mean ± *SEM*. One‐way ANOVA with a Tukey post‐test was used to determine statistical significance. In a bar chart, different lowercase letters indicate significance (*p* < 0.05).

The intestine is the first line of defense against foreign pathogens, and intestinal permeability can indirectly reflect the status of the intestinal barrier. As demonstrated in Figure 4a, d‐lactic acid (DLA) and diamine oxidase (DAO) contents in the control group treated with the bacterial solution were significantly increased (*p* < 0.05) compared with the blank group. After oral administration of various R7I doses and 20 mg/kg colistin sulfate, DAO, and DLA levels in most treatment groups were significantly decreased (*p* < 0.05), with the best results in the 20 mg/kg R7I group. In addition, we reported that partial dose concentrations of R7I and colistin could raise secreted immunoglobulin A (SlgA) levels compared to the control group, without statistical significance, and the highest content in R7I‐20 group was 2.59 mg/L. In addition, R7I significantly increased total antioxidant levels and decreased MDA levels in the small intestine (*p* < 0.05, Figure S1).

Given the excellent performance of 20 mg/kg R7I, we further probed its effects on intestinal inflammation and tight junction proteins at mRNA and protein expression levels (Figure 4b,c). Compared with the blank group, the mRNA relative expression levels of pro‐inflammatory factors IL‐1β, IL‐6, and TNF‐α were significantly elevated in the control group (*p* < 0.05), while anti‐inflammatory factors IL‐4 and IL‐10 were significantly reduced (*p* < 0.05) (IL‐10 in the ileum was an exception). After being treated with 20 mg/kg R7I, the mRNA and protein relative expression levels of pro‐inflammatory factor IL‐6 were significantly lower in the ileum, and those of TNF‐α were significantly lower in the colon and ileum. Meanwhile, the mRNA and protein relative expression levels of anti‐inflammatory factors IL‐4 and IL‐10 significantly increased in the colon. These interesting results suggested that R7I might play different regulatory roles in the inflammatory factor pathway of the colon and ileum. Additionally, for tight junction proteins, the mRNA relative expression levels of Occludin and Claudin 1 in the colon of the control group were significantly reduced compared to the blank group (*p* < 0.05). However, the 20 mg/kg R7I increased the mRNA relative expression levels of Occludin and Claudin 1 in the colon without statistical significance (*p* > 0.05).

Subsequently, we visually observed the protective effects of R7I on intestine injury by determining intestine histological changes. In Figure 4d, for the ileum, the control group had a large infiltration of inflammatory cells in the field of view (red arrows), and localized areas of the mucosal layer were separated from the lamina propria (blue arrows). In the 20 mg/kg R7I group, the tissue structure was basically normalized, only with a small number of detached epithelial cells (yellow arrow). In the blank group, the morphology of the intestinal villi was intact, and the thickness of the intestinal muscularis was uniform. In the case of the colonic tissue, the intestinal villi of the control group were structurally adherent, and epithelial cells were shed (yellow arrows). The serosal layer cells were proliferated in the local area in R7I‐20 group (red arrows). The tissue structure of the blank group was basically normal. In summary, R7I‐20 group restored the normal morphology of the intestine.

#### 2.2.2 R7I had a good protective effect on the liver injury caused by *E. coli* infection

**FIGURE 5:**
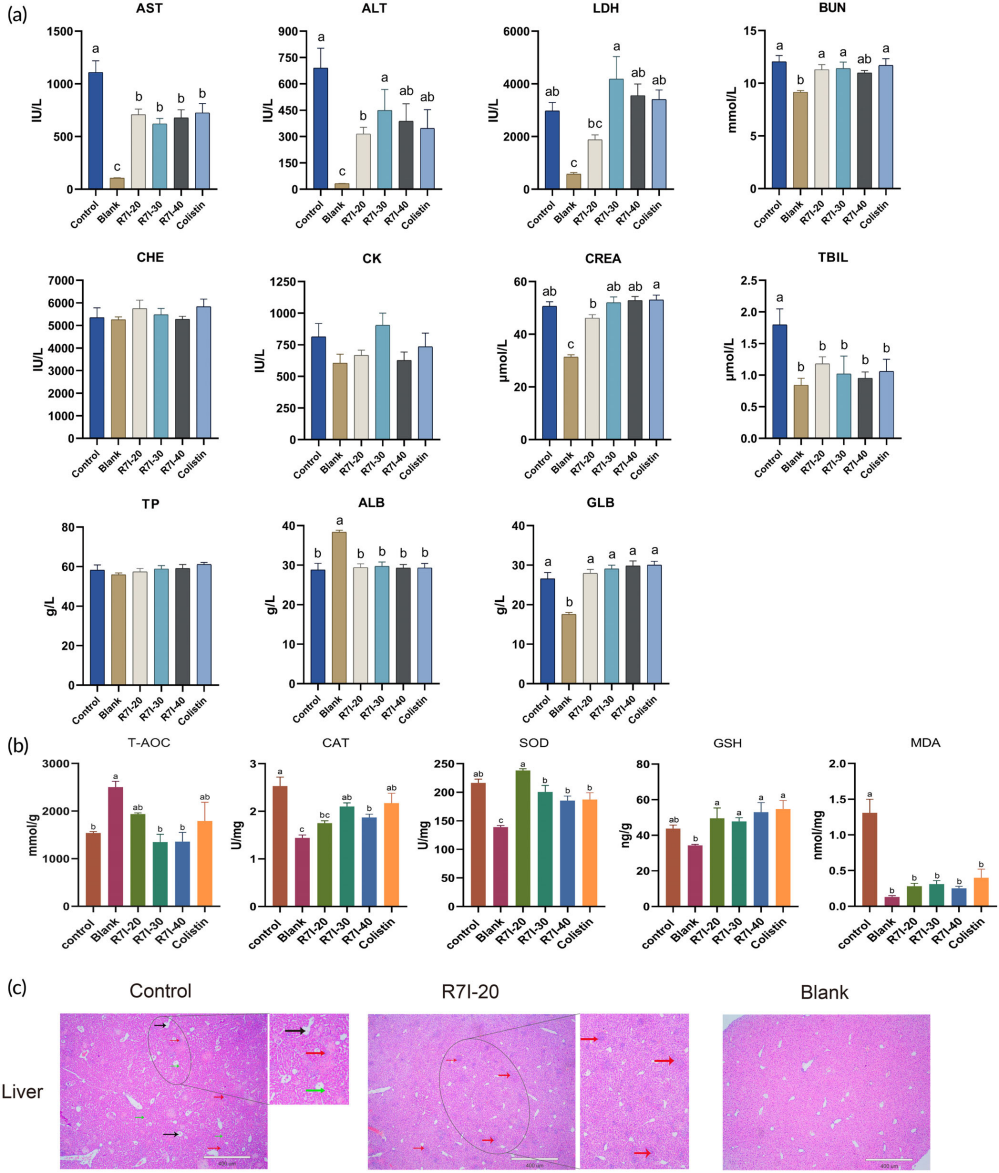
(a) Serum biochemical indices such as alanine aminotransferase (ALT), aspartate aminotransferase (AST), alkaline phosphatase (ALP), total bilirubin (TBIL), cholinesterase (CHE), creatinine (CREA), creatine kinase (CK), urea nitrogen (BUN), lactate dehydrogenase (LDH), total protein (TP), albumin (ALB), and globulin (GLB) were measured. The data was presented as mean ± *SEM* (*n* = 6–10). One‐way ANOVA with the Tukey post‐test was used to determine statistical significance. A *p* value of <0.05 was regarded as significant. (b) Antioxidant levels in the liver of mice (*n* = 6). (c) H&E‐stained histological sections of liver tissues. Scale bars, 400 μm. In the liver, black arrows stand for dilated central vein; red arrows stand for inflammatory cell infiltration; green arrows stand for vacuolated change.

The weakened intestinal mucosal barrier functions propagated and translocated numerous intestinal pathogenic bacteria, and releasing several toxic substances, such as endotoxin, would cause a systemic inflammatory response and organ injuries. Consequently, the blood biochemical indices were performed to determine whether oral R7I administration could alleviate other organ injuries in bacteria‐infected mice. As displayed in Figure 5a, liver‐associated indexes (alanine aminotransferase (ALT), aspartate aminotransferase (AST), lactate dehydrogenase (LDH), total bilirubin (TBIL), albumin (ALB) and globulin (GLB)) and kidney associated indexes (creatinine (CREA), urea nitrogen (BUN)) in the blank group were significantly different from those in the control group treated with bacterial solution (*p* < 0.05). After oral administration of various R7I doses and 20 mg/kg colistin sulfate, we found that 20 mg/kg R7I could significantly decrease AST, ALT, and TBIL levels in the serum of bacterially infected mice compared with the control group (*p* < 0.05), and relieve the elevation of LDH, CREA and CK levels, without statistical significance (*p* > 0.05). In contrast, other doses of R7I and colistin sulfate could also decrease the levels of AST, TBIL (*p* < 0.05), and ALT (*p* > 0.05), but had little effect (or even an increasing trend) on LDH and CREA levels. Given this, we preliminarily determined that R7I was more effective in protecting the liver function of bacteria‐infected mice.

The inflammation releases multiple inflammatory cytokines causing oxidative stress, further aggravating the liver injury and even promoting hepatocytes apoptosis. Consequently, attenuated oxidative stress is one of the most important measures to treat liver injury. As described in Figure 5b, the levels of malondialdehyde (MDA), a marker of oxidative stress, CAT, and SOD in the control group were significantly higher than that in the blank group, and T‐AOC levels were significantly lower than those that in the blank group, implying that bacterial infections did disrupt the disruption of antioxidant/prooxidant equilibrium in the liver. Notably, after oral administration of R7I and colistin sulfate, MDA levels in the liver significantly dropped compared with the control group. Among these, 20 mg/kg R7I could also significantly downregulate CAT levels (*p* < 0.05), increase SOD and GSH contents (*p* > 0.05), and restore T‐AOC levels (*p* > 0.05) in the liver in bacteria‐infected mice. In comparison, the antioxidant effects of other doses of R7I and colistin sulfate were slightly less.

Based on the above results, 20 mg/kg R7I showed more effective protection effects against liver injury than other treatment groups. Similarly, histological observations of livers strongly supported the protective effect of 20 mg/kg R7I. As displayed in Figure 5c, the hepatocytes in the blank group were tightly arranged and of uniform size, without evident pathological changes. However, after treatment with bacteria, the liver tissue was abnormally structured with loosely arranged tissue and dilated central veins (black arrows), inflammatory cell infiltration was observed in many parts of the tissue (red arrows), and numerous vacuolar‐like changes were visible in the field of view (green arrows). Excitingly, in the R7I‐20 group, although there remained a few inflammatory cells distributions (red arrows), the liver tissue was tightly arranged and returned to normal.

Overall, we considered that 20 mg/kg R7I had great protective effects on the liver injury caused by *E. coli* infection.

### 2.3 Effects of R7I on organ functions of the small intestine and liver

**FIGURE 6:**
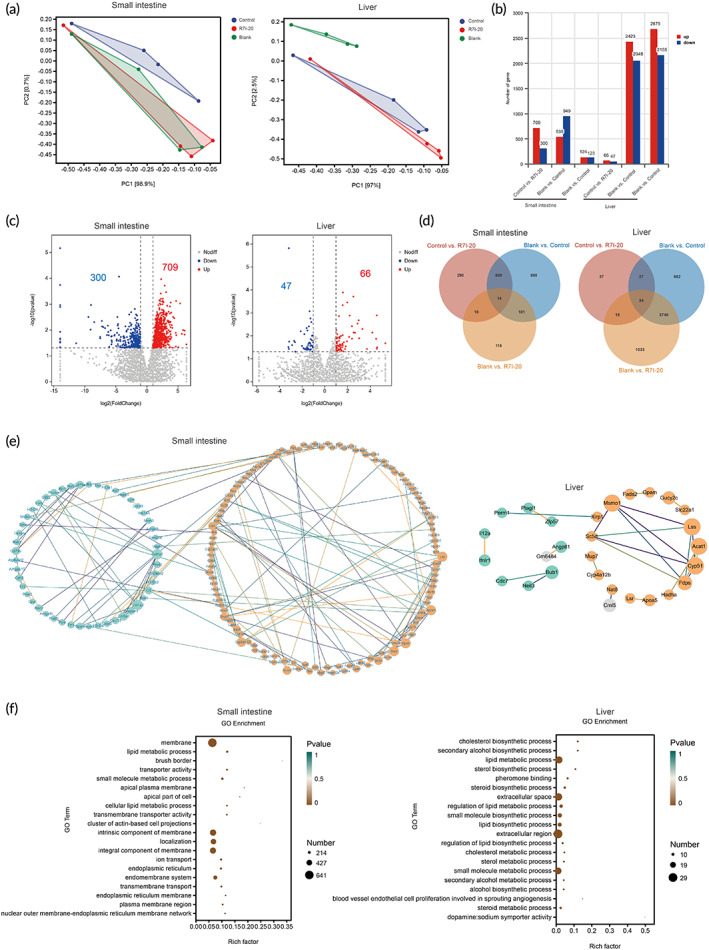
Small intestine and liver transcriptome analysis. (a) Principal component analysis (PCA); different colors indicate different groupings. (b) Statistics of differential expression results, horizontal coordinates indicate the comparison group for differential analysis, vertical coordinates demonstrate the number of differential genes, and colors indicate up or downregulation. (c) Volcano map of differential expressed genes (DEGs) in the small intestine and the liver (Control vs. R7I‐20). The horizontal coordinate is log2FoldChange, and the vertical coordinate is the significance level against 10, taking the negative logarithm of the value. The two vertical dashed lines in the graph are the thresholds for expression difference multiples; the horizontal dashed line is the significance level threshold. Colors indicate whether the gene is upregulated, downregulated, or nonsignificantly differentially expressed. (d) Venn diagram of DEGs. (e) Protein network interaction analysis (Control vs. R7I‐20). Blue indicates downregulated genes, orange indicates upregulated genes, and the combined score is indicated by the size of the circle, with the linkage representing the interaction. Disconnected nodes are not displayed in the network. (f) Top 20 terms for GO enrichment analysis of DEGs (Control vs. R7I‐20). *p* value calculated by hypergeometric distribution method (significant enrichment is defined as *p* < 0.05, *n* = 4).

Each organ function is critical in the overall health system. Digestion and absorption in the small intestine provide energy and nutrients for various life activities, while the liver is primarily responsible for material metabolism. In addition to immunity and injury examinations, the intestinal tract and liver must be examined for the bacterial attack. Below, we employed transcriptome analysis to examine functional changes in the small intestine and liver in R7I‐20 group. The sequencing data were further filtered to remove some spliced and low‐quality reads, obtaining an average number of 40,309,056 high‐quality sequence reads. The average percentage of high‐quality sequence reads in sequencing reads was 90.71%. Principal components analysis (PCA) was conducted on each sample based on the amount of expression. PCA analysis can cluster similar samples together with closer proximity, indicating higher similarity. In the case of the small intestine (Figure 6a), R7I‐20 group was similar to and overlapped with the blank group and separated from the control group (PC1 = 98.9%). For the liver, R7I‐20 group was similar to and overlapped with the control group, and they were separated from the blank group (PC1 = 97%). The Venn diagram (Figure 6d) displayed the presence of 290 unique differential genes in the small intestine in Control vs. R7I‐20 compared to the other two combinations. There were 14 differential genes common to all three combinations. Furthermore, it can be observed that the number of differential genes in Blank vs. Control is much greater than in Blank vs. R7I‐20, 5.86 times (680/116), indicating that R7I‐20 effectively reduced the number of differential genes caused by bacterial treatment. In the liver, there were 37 unique differential genes in Control vs. R7I‐20 compared to the other two combinations. There were 34 differential genes common to all three combinations. However, there was no significant difference in the number of differential genes between Blank vs. R7I‐20 and Blank vs. Control (1033/662). The volcano plot depicts A vs. B with 709 upregulated genes and 300 downregulated genes in the small intestine and 66 upregulated genes and 47 downregulated genes in the liver (Figure 6c). Figure 6e depicts the protein interaction network analysis for Control vs. R7I‐20, with blue representing downregulated genes and orange representing upregulated genes, and the combined score is shown by the size of circles, with connecting lines reflecting the interactions. Overall, R7I‐20 group had a greater effect on the small intestine than the liver.

We next performed functional classification of differential genes in each combination by Gene ontology (GO) and Kyoto encyclopedia of genes and genomes (KEGG) enrichment analyses to better understand the specific roles played by R7I, focusing on control and R7I‐20 groups. As revealed in Figure 6f, in the cellular components of the small intestine, R7I‐20 (Control vs. R7I‐20) upregulated membrane and membrane‐associated components (GO:0016020, GO:0031224, GO:0016021, and GO:0016020), cell membrane network (GO:0005783), collection of membrane structures involved in intracellular transport (GO:0012505), continuous membrane network of outer nuclear and endoplasmic reticulum membranes, and microvilli (GO:0005903). Among the biological processes involved, R7I‐20 upregulated lipid and small molecule metabolic processes (GO:0006629 and GO:0044281) and transmembrane transport (GO:0055085 and GO:0006811). In molecular function, R7I‐20 upregulated transmembrane transporter activity (GO:0022857) and the direct movement of substances (e.g., macromolecules, small molecules, and ions) within cells, outside cells or between cells (GO:0005215). The KEGG enrichment analysis of the small intestine was presented in detail in Table S2. Figure 6f displayed that in the cellular components of the liver R7I‐20 upregulated the collection of membrane‐like structures involved in intracellular transport (GO:0005615, GO:0005789, GO:0042175, and GO:0005783). In biological processes, R7I‐20 primarily facilitated the synthesis and metabolic processes of lipid‐like substances, including cholesterol, steroids (GO:0006629, GO:0019216, GO:0008610, GO:0046890, and GO:0006694), small molecule biosynthesis, and metabolic processes (GO:0044283 and GO:0044281). The liver KEGG enrichment analysis was presented in detail in Table S3. These results indicated that R7I‐20 group restored the normal physiological functions of the liver and intestine to a certain extent, ensuring the normal functioning of the organism. Pathway analysis of the small intestine and liver of the control and R7I‐20 groups as compared to the blank group were displayed in Tables S4–S7.

### 2.4 Regulatory effects of R7I on intestinal microflora and metabolites

#### 2.4.1 R7I promotes homeostasis of intestinal flora

**FIGURE 7:**
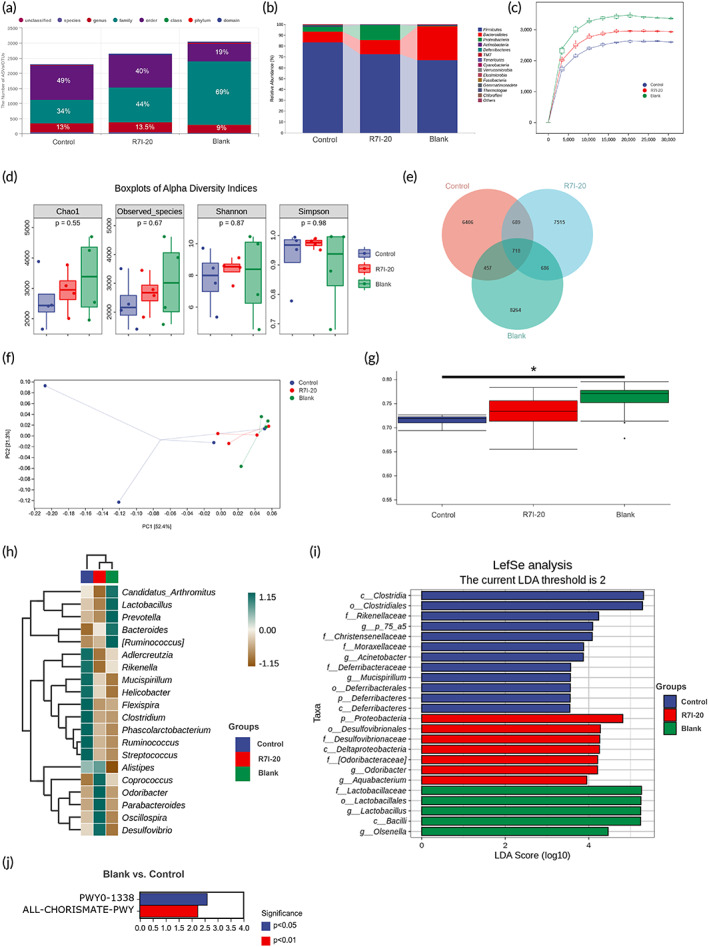
Gut microbiota analysis by 16S rRNA sequencing. (a) Statistics on the number of taxonomic units of species composition. (b) Analysis of taxonomic composition at the phylum level. (c) The rarefaction curve, with the horizontal coordinate being the leveling depth and the vertical coordinate being the median value of the alpha diversity index calculated 10 times versus the box plot. Comparing the number of ASV/OTU in different samples at the same sequencing depth (Blank > R7I > Control). (d) Alpha diversity index. In this process, Chao1 and observed species index were used to represent richness, and Shannon and Simpson's indexes were used to represent diversity. Significance was tested using Kruskal–Wallis rank‐sum test and Dunn's test as a post hoc test. (e) ASV/OTU Venn diagram. (f) Orthogonal projections to latent structures discriminant analysis (OPLS‐DA). Each dot represents a sample, with different colored dots indicating different groupings. (g) Analysis of differences between groups (analysis of similarities, *p* < 0.05). (h) Genus level random forest analysis. From top to bottom, species were of decreasing importance to the model. (i) LDA effect size analysis. The significance passed the Kruskal–Wallis test. (j) Differential analysis of metabolic pathways was predicted using PICRUSt2. Using the metagenomeSeq method, the horizontal coordinate indicates log2 (fold change). Only statistically significant differences were found between the control and blank groups, and the control group was upregulated. Each set of data contained four biological replicates.

Intestinal flora can influence intestinal health, especially in inflammatory bowel disease, irritable bowel syndrome, and other intestinal disorders. Following that, the cecal contents 16 S rRNA gene was sequenced to explore intestinal flora changes. Twelve samples from three groups (Control, R7I‐20, and Blank) were sequenced, and 1,058,307 sequences matched the forward and reverse primers in the original data, with an average of 88,192 per sample. A total of 592,473 high‐quality sequences were generated by Dada2 method after denoising and clustering. Subsequently, species taxonomic annotation was conducted. The average sequence length of the entire sample was 415 bp. The taxonomic annotation unit disclosed that the control group (*E. coli* infection) reduced the proportion of ASVs at the family level while increasing them at the order level (Figure 7a). As displayed in Figure 7e, ASVs in three groups (Control, R7I‐20, and Blank) were 8270, 9608, and 10,125, respectively. There were at least 2550 ASVs shared by the two groups, of which 718 were shared by the three groups. Each of the three groups had 6406 (Control), 7515 (R7I‐20), and 8264 (Blank) unique ASVs. In Figure 7b, the proportion of Control and R7I‐20 of *Bacteroidetes* decreased to 9.95% and 13.04%, respectively, while that of Blank was 31.45%. *Proteobacteria* was increased in R7I‐20 (13.43%), compared with Control (4.76%) and Blank (0.59%) groups. *Firmicutes* in Control increased to 83.15%, and the ratio of *Firmicutes* to *Bacteroidetes* increased.

Figure 7c illustrated the rarefaction curve in which the alpha diversity index of the sample tended to be flat with the increased sequencing depth. The alpha diversity index was arranged from large to small as Blank, R7I‐20, and Control. Chao1 and observed species index had no significant difference in characterization richness between the three groups, and R7I‐20 was between Control and Blank groups (Figure 7d). Similarly, Shannon and Simpson indexes showed no significant difference in uniformity between the three groups. In Figure 7f, orthogonal partial least squares discriminant analysis (OPLS‐DA) was employed to analyze the difference in species abundance composition at the species level. The results revealed that the top two principal components accounted for 52.4% and 21.4% of the variation in the total data, respectively. The distance of sample centers between Control and Blank was 6.14 times greater than that between R7I‐20 and Blank, primarily due to the projection on PC1. Analysis of similarities (Anosim) was utilized to analyze the differences in species composition between groups. As described in Figure 7g, the difference between Control and Blank groups was significant (*p* = 0.02).

The random forest analysis indicated the top 20 genera in relative abundance at the genus level for the three groups (Figure 7h). After that, all classification levels were analyzed simultaneously by LDA effect size (LEfSe). In Figure 7i, when the LAD score was >4, the difference advantage of Control groups was *Clostridia* and *Clostridiales*, the difference advantage of R7I‐20 group was *Proteobacteria*, and the difference advantage of the Blank group was *Lactobacillaceae*, *Lactobacillales*, *Lactobacillus*, and *bacillus*. Only PWY0‐1338 (*p* < 0.05) and ALL‐CHORISMATE‐PWY (*p* < 0.01) pathways were upregulated in the Control group compared with the Blank group in the differential analysis of predicted metabolic pathways (Figure 7j). The species composition of metabolic pathways indicated that both pathways were caused by the increase of unclassified *Enterobacteriaceae* in Control group. However, R7I‐20 indicated no difference from the other two groups.

#### 2.4.2 R7I reduces the accumulation of harmful metabolites in the gut

Intestinal flora changes inevitably affected the composition of metabolites, which in turn were closely linked to the health status of the organism. Therefore, it was necessary to understand metabolite changes in the small intestine. Raw data were converted to the common (mz.data) format by Agilent Masshunter Qualitative Analysis B.08.00 software (Agilent Technologies, USA). In R software platform, XCMS program was used in peak identification, retention time correction, and automatic integration pretreatment. Following that, the data were subjected to internal standard normalization. Visualization matrices containing sample name, m/z‐RT pair, and peak area were obtained. A total of 2425 and 6580 features were acquired in positive and negative modes, respectively. After qualitative analysis, data matrices were imported into R, followed by multivariate analysis, focusing more on Control vs. R7I‐20.

**FIGURE 8:**
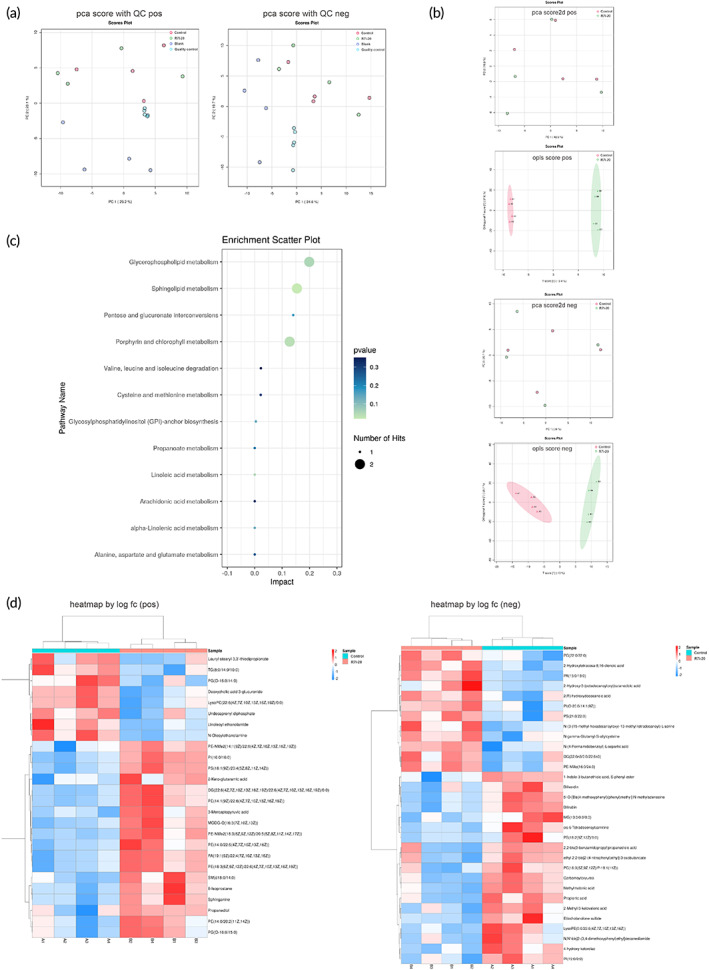
Small intestine metabolite assay. (a) The overall PCA scores are plotted with quality control (QC), positive, and negative metabolites. (b) The multivariate statistical method PCA and orthogonal partial least squares discriminant analysis (OPLS‐DA) was used to analyze the control and R7I‐20 groups. (c) Analysis of differential metabolite pathway enrichment (Control vs. R7I‐20). (d) Differential metabolite heatmaps (Control vs. R7I‐20). Pos, positive; neg, negative. Each group contained four biological replicates.

We used PCA modeling methods to examine the aggregation degree of QC samples (Figure 8a). In this project, differential metabolites were screened out by variable importance in the projection (VIP) value of orthogonal projections to latent structures discriminant analysis (OPLS‐DA) model (VIP > 1) and independent sample *t* test (*p* < 0.05) in Figure 8b. The detailed data in Control vs. R7I‐20 was presented in the differential metabolite heat map in Figure 8d. Metabolites analysis with significant differences is summarized in Tables S8 and S9 (Control vs. R7I‐20). Among them, propionic acid, methylmalonic acid, and 2‐methyl‐3‐ketovaleric acid were significantly increased in the control group. In R7I‐20 group, PS (18:1/20:4), PE‐NME2 (18:3/20:5), PE (18:3/22:6), PE (14:0/22:5), PI (16:0/16:0), and PE‐NME (16:0/24:0) were significantly increased (*p* < 0.05). Therefore, R7I‐20 group reduced the accumulation of harmful organic acid metabolites and increased lipid and glycerophospholipid metabolites. The differential metabolites were mapped to KEGG ID by the online software MetaboAnalyst. The differential metabolite pathway enrichment analysis is presented in Figure 8c. Glycerophospholipid metabolism, sphingolipid metabolism, pentose and glucuronate interconversions, porphyrin, and chlorophyll metabolism were significantly enriched in Control vs. R7I‐20. The significantly different metabolites in the small intestine of the control and R7I‐20 groups compared with the blank group are displayed in Tables S10–S13.

## 3 MATERIALS AND METHODS

### 3.1 Ethics approval and consent to participate

The protocols used in this experiment were approved by the Northeast Agricultural University Institutional Animal Care and Use Committee. All the Animal care and treatment were complied with the standards described in the “Laboratory Animal Management Regulations” (revised 2016) of Heilongjiang Province, China.

### 3.2 Synthesis and characterization of peptides

R7I (IRPI IRPI IRPI IRPI IRPI IRPI IRPI‐NH_2_) and fluorescein isothiocyanate (FITC)‐labeled R7I were synthesized by GL Biochem Corporation (Shanghai, China), and it was determined by matrix‐assisted laser desorption/ionization time‐of‐flight mass spectrometry (MALDI‐TOF MS; Linear Scientific Inc.). The peptide purity (>95%) and retention time were tested by reversed‐phase high‐performance liquid chromatography (HPLC).

### 3.3 Stable metabolism of R7I in vivo

The C57BL/6 male mice aged 6 weeks were provided by Liaoning Changsheng Biotechnology Co., Ltd., China and euthanized at 10:00 am. The serum was collected and mixed with R7I (v/v = 4:1) to give a final peptide concentration of 1.28 mM. The stomach (cut in half) and the contents of the entire small intestine of the mice were collected and placed in a sterile eppendorf tube. Sterile PBS was added to gastric tissue (w/w = 1:1, pH = 2.0) and small intestine contents (w/w = 1:9, pH = 7.0), and mixed thoroughly by shaking. The mixture was centrifuged to obtain the supernatant as gastric juice and small intestine juice. R7I was dissolved with the corresponding pH PBS. The prepared gastric and small intestine fluids were then mixed separately with R7I (v/v = 1:1) to give a final peptide concentration of 1.28 mM. Melittin was used here as a control. The peptide mixture was incubated in a 37°C incubator for 1, 4 and 8 h. Finally, the minimum inhibitory concentrations (MICs) test was performed to verify peptide activity with *Escherichia coli* 25922.^18^

Subsequently, the C57BL/6 male mice (6 weeks old) were fasted for 12 h before treatment, and the blank group was instilled with saline (200 μl), while the experimental group was instilled with 20 mg/kg of body weight FITC‐R7I (200 μl) to start the clock. Specimens were collected at 1 h, 4 h, and 8 h (each organ and each mid‐gut), respectively. The blank group served as a control, and all samples were photographed under a fluorescent microscope set up with a uniform light source.

### 3.4 The enteritis model and experimental design

C57BL/6 male mice that were 6 weeks old were provided by Liaoning Changsheng Biotechnology Co., Ltd., China. All mice (20.00 ± 2.00 g, 6 weeks old) were randomly divided into 6 groups (*n* = 12), and they were fed adaptively for 3 days. During the whole experiment, mice can get clean water and a laboratory standard diet ad libitum, under controlled environmental conditions (40%–60% relative humidity, 25 ± 2°C temperature; lighting cycle, 12 h/d). The standard lab diet contained 18% crude protein, 4% crude fat, 5% crude fiber, 8% ash, and 10% moisture.

In pre‐experiments, we had observed that lower peptide concentrations did not make a significant difference. The mice were divided into six groups randomly: (1) the control group (*E. coli* + normal saline); the administration group (*E. coli* + R7I) is set to three concentration gradients: (2) 20 mg/kg, (3) 30 mg/kg, and (4) 40 mg/kg of body weight; (5) the antibiotic group (*E. coli* + colistin sulfate), 20 mg/kg of body weight; and (6) the blank group with only normal saline (Figure 2b).

Based on the results of the pre‐experiment, we determined the appropriate concentration of the bacteria. The strain *E. coli* ATCC 25922 frozen at −20°C was incubated overnight in LB medium (37°C, 220r). The bacterial broth was transferred to a new LB medium at a ratio of 1:100 for 3–5 h to reach the logarithmic growth period. The bacterial precipitate was centrifuged and washed three times with saline to adjust the colony count to 2.0 × 10^9^ CFU/ml.

The detailed processing process was shown in Figure 2a. The blank group was gavaged with 200 μl of saline, and all other groups were gavaged twice with *E. coli* ATCC 25922 (200 μl) once a day (d1‐2). R7I at different concentrations and colistin sulfate were given twice (12 h intervals) on the d3 and once on d4‐5 by oral administration. Correspondingly, an equal amount of saline was given to the blank and control groups in the same way. On day 6, all mice were euthanized by cervical dislocation under full anesthesia with isoflurane.

### 3.5 Collection of blood, tissues, and organs samples

The collected blood was centrifuged for 10 min at 3000 rpm at 4°C. The supernatant was taken and stored at −80°C for subsequent testing. The liver and part of the middle ileum were cut and placed in precooled 4% paraformaldehyde for hematoxylin eosin (H&E) staining. The Liver, small intestine, colon, and cecum contents were stored separately in cryogenic vials at −80°C.

### 3.6 Blood biochemical analysis

Serum biochemical indices of alanine aminotransferase (ALT), aspartate aminotransferase (AST), alkaline phosphatase (ALP), total bilirubin (TBIL), cholinesterase (CHE), creatinine (CREA), creatine kinase (CK), urea nitrogen (BUN), lactate dehydrogenase (LDH), total protein (TP), albumin (ALB), and globulin (GLB) were measured by the automatic biochemical analyzer (Roche, Cobus‐Mira‐Plus, Roche Diagnostic System Inc., Basel, Switzerland).

### 3.7 Histological analysis

Histological analysis was performed by hematoxylin–eosin staining (H&E). In brief, liver, ileum, and colon of mice were fixed overnight with 4% paraformaldehyde‐PBS, dehydrated, and embedded in paraffin blocks. Then the samples were cut into 5 μm sections that were deparaffinized and hydrated and then stained with H&E for histological analysis.

### 3.8 Antioxidant function of the liver and small intestine

Total antioxidant capacity (T‐AOC), superoxide dismutase (SOD), glutathione peroxidase (GSH‐Px), malondialdehyde (MDA), and catalase (CAT) enzyme activities in the liver were determined using commercially available kits (Nanjing Jiancheng Bioengineering Institute, Nanjing, China) according to the manufacturer's instructions.

### 3.9 The intestinal barrier

To detect the content of diamine oxidase (DAO), d‐lactic acid, and secretory immunoglobulin A (SlgA) in serum, using mouse ELISA kit instruction (Alphabio, Tianjin, China).

### 3.10 RNA extraction and real‐time quantitative PCR

Briefly, total RNA was extracted with Trizol, and then RNA was reversely transcribed into cDNA after genomic DNA was removed by a two‐step method according to the instructions of the kit. SYBR Green method was used to analyze the mRNA expression of specific primers for different genes. The reagents and kits were purchased from Nanjing Vazyme Biotech Co., Ltd., China. Subsequently, real‐time PCR was performed using CFX Connect (Bio‐Rad, Hercules, CA, USA). Primer 5.0 software was used to design primers for specific genes (Table S1). The internal control gene was β‐actin. The relative abundance of target genes was calculated using the 2^−ΔΔCt^ approach. The expression of target gene mRNA in the control group was taken as the baseline relative to the treatment group (i.e., fold‐change).

### 3.11 Assessment of protein expression by western blot analysis

Total protein from ileum and colon tissues was separated using commercial protein extraction reagents (Beyotime, Shanghai, China). Reducing SDS‐PAGE electrophoresis was conducted to separate 40 μg protein, which was then transferred to PVDF membranes (Millipore, Billerica, MA, USA). Afterward, membranes were blocked with 5% skimmed milk for 2 h in Tris‐Tween saline buffer, followed by incubation using primary antibodies. The HRP‐conjugated secondary antibodies were subsequently incubated for 2 h at room temperature. The Alpha Imager 2200 software (Alpha Innotech Corporation, San Leandro, CA, USA) was employed to develop protein blots. Lastly, protein signals were quantified digitally and normalized to the relative expression of β‐actin. ImageJ software (National Institutes of Health, MD, USA) was used to determine the band density. See Supporting Information for antibody information.

### 3.12 RNA‐Seq analysis

The sequencing service was provided by Shanghai Personal Biotechnology Co., Ltd., China. Briefly, total RNA was extracted from the liver and ileum. The cDNA libraries were constructed and then sequenced using the Illumina NovaSeq sequence platform. A total of 24 samples with 4 biological replicates per treatment were available for RNA‐seq analysis. The filtered Reads were matched to the mouse reference genome (Mus_musculus.GRCm39.dna.primary_assembly.fa) using HISAT2 (http://ccb.jhu.edu/software/hisat2/index.shtml) software. Differential analysis of gene expression was performed using DESeq, and the conditions for screening differentially expressed genes were: expression difference ploidy |log_2_FoldChange| > 1 and significance *p* value <0.05. Based on the results of differential gene expression analysis, PPI pairs containing the differential genes with a score > 0.95 were screened using the STRING database (https://string-db.org/cgi/input.pl), and then protein interaction networks were mapped. GO enrichment analysis and KEGG enrichment analysis were performed using topGO and cluster profiler, respectively. In the analysis, the gene list and gene number of each term were calculated using the differential genes annotated by GO term, and the gene list and gene number of each pathway were calculated using the differential genes annotated by KEGG pathway, so as to determine the main biological functions of differential genes.

### 3.13 Metabolites analysis

The metabolites in small intestine (ileum portion) of control group, R7I‐20 group, and blank group were detected. About 50 mg of each sample was weighted out and 400 μl methanol (containing 5 μg/ml 2‐chloro‐l‐phenylalanine as internal standard) was added to it. The mixture was mixed by a vortex mixer for 1 min and homogenized for 3 min at 60 Hz twice. Then the mixture was centrifuged at 13,000 rpm, 4°C for 10 min. The supernatant was transferred to sampler vials for detection. An in‐house quality control (QC) was prepared by mixing an equal amount of each sample. Detailed information on the working model can be found in Supporting Information.

### 3.14 16S rRNA amplicon sequencing

Then, we determined the flora of cecum contents. The V3‐V4 hypervariable regions of 16 S rDNA were amplified (forward: ACTCCTACGGGAGGCAGCA, reverse: GGACTACCAGGGTATCTAATCCTGTT), and the amplified products were then sequenced by the Illumina platform. DADA2 method mainly included primer removal, quality filtering, denoising, splicing, and clustering. Greengenes database (Release 13.8, http://greengenes.secondgenome.com/) was selected for species annotation. Sequencing service was provided by Shanghai Personal Biotechnology Co., Ltd., China. The data were analyzed by using the free online platform Personalbio GenesCloud (https://www.genescloud.cn/).

### 3.15 Statistical analysis

Results are reported as means ± standard errors of the mean (*SEM*) and evaluated with one‐way analysis of variance (ANOVA). The Tukey test was used to detect differences among treatments. The SPSS V25 (SPSS Inc., Chicago, IL, USA) software was used for all statistical analyses. A *p* value of <0.05 was considered statistically significant. All data was visualized using Graphpad Prism 8.0 (Graphpad Inc., CA, USA).

## 4 DISCUSSION

With over one million people worldwide suffering from inflammatory bowel diseases such as Crohn's disease and ulcerative colitis, the need for novel antibiotics and treatments is pressing.^19^ For any new drug, developing new antibiotics requires a balance between optimizing efficacy in killing bacteria and minimizing toxicity to human or animal hosts, as well as establishing suitable physicochemical properties to allow appropriate delivery and pharmacokinetics.^20^, ^21^ Therefore, we have thoroughly described in detail the actual in vivo efficacy of the anti‐enzymatic peptide R7I.

Before this, for serum and low pH gastric juice, R7I function was hardly affected. In the small intestinal fluid, R7I activity was only slightly disturbed and was far superior to that of the melittin. Unused data revealed that melittin was inactivated even after incubation with the small intestinal fluid for 1 min. This strongly suggested that R7I was resistant to enzymatic hydrolysis and could be administered orally.

Following that, we examined the function of the intestinal barrier. Intestinal permeability embodies digestive and absorptive functions, as well as the barrier function. Serum d‐lactate reflects the degree of intestinal mucosal damage and permeability, and DAO reflects the integrity and degree of damage to the physical barrier of the gut.^22^ R7I and Colistin significantly reduced d‐lactic acid and DAO, mitigating damage to the intestine and mucosa, and lower R7I‐20 concentrations presented better results. SIgA is critical in protecting mucosal surfaces against pathogens and maintaining homeostasis with commensal flora.^23^, ^24^, ^25^ Additionally, SIgA contributes to reducing pathogen‐mediated pro‐inflammatory responses in epithelial cells.^26^ R7I‐20 and Colistin increased SIgA content without statistical significance, and the positive effect on SIgA was diminished with increasing R7I concentrations. These findings suggested that high R7I concentrations were the inappropriate choice.

Subsequently, the gut was assayed for inflammatory factors and tight junction proteins. In the inflammatory response, IL‐10 suppresses inflammatory cell activation, migration, and adhesion by downregulating the expression of major histocompatibility complex class II (MHC II) on the surface of monocytes, reducing their antigen‐presenting effects and downregulating T‐lymphocyte activity.^27^ Meanwhile, IL‐10 inhibits the synthesis and release of inflammatory factors.^28^ IL‐4 exhibits immunomodulatory effects on B‐lymphocytes, T‐lymphocytes, mast cells, and macrophages.^28^, ^29^ As an inflammatory cytokine produced by macrophages or monocytes during acute inflammation, tumor necrosis factor α (TNF‐α), is responsible for various intracellular signaling events leading to necrosis or apoptosis, and IL‐6 is a member of the pro‐inflammatory cytokine family that induces the expression of various proteins associated with acute inflammation.^30^, ^31^, ^32^, ^33^ We found that R7I‐20 decreased pro‐inflammatory factors IL‐6 and TNF‐α in the ileum, while in the colon, R7I‐20 increased anti‐inflammatory factors IL‐4 and IL‐10 and decreased TNF‐α. These findings suggested a regulatory role for R7I‐20 in the intestinal inflammatory pathway. Moreover, tight junction is an important form of intercellular junction and the most critical structure forming the mechanical barrier of mucosa. R7I‐20 slightly increased Claudin 1 and Occludin expressions in the ileum and colon, without statistical significance.

Regarding the relevant indicators of serum, it can be found that after the mice were administrated twice with the bacterial liquid, the violent increase of ALT and AST indicated that the liver was seriously damaged, and unexpected elevation of lactate dehydrogenase represents liver disease acute hepatitis or certain malignancies.^34^ The liver has an essential role in TBIL metabolism, including the uptake, binding, and excretion of unbound bilirubin in the blood by hepatocytes.^35^, ^36^ R7I and colistin provided some relief from ALT, AST, and TBIL and mitigated liver damage from *E. coli* infection. In addition, R7I reduced LDH and CREA levels at low concentrations, but R7I and Colistin did not clearly improve BUN, while CREA and BUN concentrations indirectly reflected glomerular filtration function.^37^ CK is required to pass through the liver and be excreted by the bile, and any abnormality in the whole process can cause elevated alkaline phosphatase levels.^38^ R7I‐20, 40 was found to slow down the rise of CK.

Regarding liver antioxidant function, CAT levels in the control group were significantly higher than in the blank group. In contrast, CAT levels in R7I‐20 group were similar to the blank group status, indicating abnormal activation of the hepatic peroxisome pathway leading to abnormal CAT levels. No significant improvement was found for GSH and SOD. Bacterial treatment resulted in a decrease in T‐AOC and a slight increase in R7I‐20. In addition, oxygen radicals act on lipids to cause peroxidation, the end product of which is MDA, which causes cross‐linking and polymerization of proteins, nucleic acids, and other living macromolecules, damaging cell structure and function.^39^ All three concentrations of R7I and colistin significantly reduced MDA levels. Overall, R7I mitigates the oxidative process in the liver and plays a protective role. In paraffin sections, R7I‐20 treatment reduced the central venous dilatation in the liver, and the tissue was more compact, but a small amount of sexual cell infiltration remained present. R7I‐20 reduced the inflammatory cell infiltration in the ileum, and a small amount of cell proliferation remained in the colon. This indicates that R7I was effective in relieving damage to the liver and intestinal tract.

According to transcriptome analysis, R7I‐20 treatment greatly reduced the number of small intestinal differential genes by 5.86‐fold (680/116) compared to the control group, but there was no difference in the liver, indicating that *E. coli* damage in the gut can be treated quickly and effectively. PCA revealed similarities between the R7I‐20 group and the blank group in the intestine, which was a positive finding but not in the liver (Figure 6a). This may be closely related to the route of oral administration and metabolism. In the small intestine enrichment analyses, R7I‐20 enhanced membrane‐associated and microvilli‐associated processes and facilitated transmembrane transport activities such as signaling and substance exchange between cell membranes. R7I‐20 group greatly improved protein and fat digestion and absorption, as well as bile secretion pathways. The specific genes are displayed in Table S2. The family of solute carriers (SLC), which plays a crucial part in the physiological process of moving from cellular uptake of nutrients to uptake of drugs and other xenobiotic compounds, is one of the most upregulated membrane transporter proteins. SLC transporters were responsible for transporting numerous molecules, including nutrients, metabolites, exogenous substances (e.g., phytochemicals), small molecule drugs, and metal ions, implying that SLCs were involved not only in key physiological processes, such as intestinal nutrient absorption but also in specific cellular tasks.^40^, ^41^ In addition, diacylglycerol acyltransferase (DGAT) is involved in forming of triacylglycerols and is linked related to intestinal fat absorption, regulating plasma triglyceride concentrations, fat storage in adipocytes, and energy metabolism in muscle.^42^ Furthermore, ABCG5 and ABCG8 form an obligate heterodimer that is critical in the selective transport of dietary cholesterol into and out of intestinal cells, as well as the selective excretion of sterols into bile by the liver.^43^ However, cholesterol is the only sterol synthesized by acetyl coenzyme A and utilized by mammals, and since few cells can metabolize cholesterol, its removal by bile and intestinal secretions is essential to maintaining homeostasis.^44^, ^45^ In summary, R7I ameliorated intestinal physiological dysfunction caused by bacterial invasion. In the liver, we stated that R7I mainly upregulated steroid biosynthesis, fatty acid degradation, and glutathione metabolism (Table S3). It has been revealed that NAT8 overexpression or downregulation prevented or exacerbated H_2_O_2_‐induced apoptosis, respectively.^46^ SC5D catalyzes the synthesis of 7‐dehydrocholesterol from lathosterol and impaired activity also leads to cholesterol deficiency, resulting in lathosterol accumulation.^47^ NAT8 and SC5D upregulation in the R7I‐20 group maintained normal liver function.

Following that, R7I significantly reduced the increase of negative metabolites in the control group while upregulating some positive metabolites in the metabolite analysis of the small intestine. Research has indicated that mouse models were transfected with Crohn's disease‐associated adherent invasive *Escherichia coli* (AIEC), in which elevated intestinal propionic acid levels led to AIEC recovery and remarkably increased toxicity.^48^ Therefore, exposure to propionic acid leads to AIEC resistance and increased virulence.^48^ Enzyme inhibition by methylmalonic acid may lead to various metabolic disorders and inhibit mitochondrial energy generation in mammals deficient in vitamin B‐12.^49^ In addition, methylmalonic acid accumulation was a major feature of methylmalonic aciduria.^50^ 2‐Methyl‐3‐ketovalic acid was a known pathological metabolite associated with propionic acidosis, especially during ketoacidosis.^51^ These negative metabolites were significantly elevated in the control group compared to R7I‐20 group. Phosphatidylserine (PS (18:1/20:4)) was a multifunctional bioactive lipid and an essential anionic phospholipid for cell membranes playing important roles in physiological processes such as apoptosis, inflammation, and coagulation.^52^, ^53^ Recent data have revealed the potential of phosphatidylserine to protect against atherosclerosis, reflecting its ability to suppress inflammation, regulate blood coagulation, and enhance high‐density lipoprotein (HDL) function.^54^ PI (16:0/16:0), a phosphatidylinositol, was an important lipid, a key membrane component and a participant in important metabolic processes, as well as a major source of arachidonic acid in animal tissues.^55^, ^56^ R7I‐20 group greatly increased the levels of these two lipids in the small intestine and reduced the amount of harmful organic acids compared to the control group, protecting intestinal health. Moreover, R7I‐20 group increased glycerophospholipid metabolites, including dimethylphosphatidylethanolamine (PE‐NMe2 (18:3/20:5)) and phosphatidylethanolamine (PE (18:3/22:6), PE (14:0/22:5)), jointly promoting glycerophospholipid metabolism and sphingolipid metabolism. Compared to the blank group, metabolites such as p‐Cresol glucuronide, leukotriene C4, various forms of acylcarnitine, lithocholic acid, and cortisol were significantly increased in the small intestine of the control group (Tables S10 and S11). In contrast, metabolites such as cortisol, cholestane‐3,7,12,25‐tetrol‐3‐glucuronide, palmitoyl glucuronide, trans‐2‐dodecenoylcarnitine, and rock bile acid were significantly upregulated in R7I‐20 group (Tables S12 and S13). Numerous diseases have been described that cause disruptions in energy production and intermediate metabolism in organisms, characterized by the production and excretion of unusual acylcholines.^57^ P‐cresol is a neurotoxic and nephrotoxic carcinogenic aromatic substance produced by gut microbe fermentation, and it is the precursor of the prototypical protein‐bound uremic toxin p‐cresol sulfate (p‐CS).^58^, ^59^ Glucuronidation is used to assist in the excretion of toxic substances, drugs, or other substances that cannot be used as an energy source.^60^, ^61^ The glucuronic acid is attached to the substance through a glycosidic bond, and the resulting glucuronide has a much more water soluble than the original substance and is eventually excreted by the kidneys.^60^

Numerous clinical investigations have demonstrated that inflammation alters gut microbes and their metabolites, and that the affected gut and gut microbes, in turn, stimulate immune responses and metabolic activity, leading to chronic inflammation.^62^, ^63^, ^64^, ^65^ From the results, *E. coli* infestation resulted in a noteworthy reduction in species composition differences (Control vs. Blank), with Control being much further away from Blank group than R7I‐20 in OPLS‐DA. In contrast, R7I‐20 did not cause a decrease in the composition and abundance of the intestinal flora due to its attributed antimicrobial activity, possibly linked to the lower concentration. The protective effect of R7I‐20 on the stability of intestinal flora was reflected in the taxonomic annotation unit of the species, the rarefaction curve, Chao1, and the total number of ASVs. Some studies have disclosed that colonization by clostridia is particularly deleterious. Clostridia were involved in developing of necrotizing enterocolitis (NEC), the dominant bacterium in the control group.^66^ In R7I‐20 group, there remained the threat of *Desulfovibrio* in the *Proteobacteria* phylum, but another dominant strain, *Odoribacteraceae*, has been revealed to efficiently synthesize isoalloLCA.^67^ The isoalloLCA was a special bile acid that fights many infections such as *Clostridium difficile* and *Enterococcus faecalis*.^67^ It was a counter to the disruption of the homeostatic balance of the intestine. In contrast, *Lactobacillus* was mainly present in the blank group, promoting digestion and inhibiting spoilage multiplication and pathogenic bacteria in the intestine.

In the analysis of predicted metabolic pathway differences, we also identified pathway alterations caused by unclassified *Enterobacteriaceae*. PWY0‐1338 (MetaCyc Pathway: polymyxin resistance) was upregulated in the control group. Polymyxins are produced by Gram‐positive bacterium *Paenibacillus polymyxa* and can be selectively toxic to Gram‐negative bacteria by interacting with phospholipids, particularly lipid A, to disrupt the structure of bacterial cell membranes.^68^ Some Gram‐negative bacteria, specifically *Salmonella typhimurium* and *Escherichia coli*, can become resistant to polymyxin by modifying their lipid A structure by the attaching of 4‐amino‐4‐deoxy‐l‐arabinopyranose (l‐Ara4N) groups to one or more phosphate groups.^69^ In addition, the control group significantly upregulated ALL‐CHORISMATE‐PWY (MetaCyc Pathway: superpathway of chorismate metabolism). Chorismate is the principal common precursor of the aromatic amino acids l‐tryptophan, l‐tyrosine, and l‐phenylalanine, as well as the essential compounds 5, 6, 7, 8‐tetrahydrofolate, ubiquinone‐8, menaquinol‐8 and enterobactin (enterochelin). Enterobactin is a catecholate siderophore produced almost exclusively by *Enterobacteria*, although it has been reported in some *Streptomyces* species. Additionally, research has proven that Enterobactin‐mediated high‐affinity iron acquisition is critically important for Gram‐negative bacterial pathogens to survive and infect the host.^70^ Evidently, R7I effectively reduced these risks, as they were undetectable in R7I‐20 group.

## 5 CONCLUSION

For *E. coli* causing adverse effects on the organism, this study revealed that R7I greatly slowed down this process. This included reducing inflammatory factors, maintaining intestinal barrier function, facilitating small intestinal digestion and absorption and hepatic fatty acid metabolism, reducing negative organic acid metabolites while increasing lipid and glycerophospholipid metabolites, stabilizing microbial community composition and abundance, and effectively reducing the abnormal metabolic pathway by *E. coli* (polymyxin resistance, superpathway of chorismate metabolism). In general, R7I exhibited good anti‐enzymatic stability and presented potential application prospects in treating bacterial infection‐associated inflammatory bowel disease. These efforts and results provided a thorough basis and reference for advancing orally administered therapeutic peptides and proteins and contributing to the strategy of alternative antibiotics.

## AUTHOR CONTRIBUTIONS

**Taotao Sun:** Conceptualization (equal); data curation (lead); formal analysis (lead); methodology (lead); validation (equal); visualization (lead); writing – original draft (lead); writing – review and editing (equal). **Xuesheng Liu:** Data curation (equal); methodology (equal); project administration (equal); validation (equal). **Yunzhe Su:** Methodology (equal); project administration (equal); validation (equal). **Zihang Wang:** Data curation (equal); methodology (equal); project administration (equal); validation (equal). **Baojing Cheng:** Formal analysis (equal); methodology (equal); project administration (equal); validation (equal). **Na Dong:** Formal analysis (equal); methodology (equal); project administration (equal); validation (equal). **Jiajun Wang:** Conceptualization (equal); methodology (equal); project administration (equal); writing – review and editing (equal). **Anshan Shan:** Conceptualization (lead); formal analysis (equal); funding acquisition (lead); project administration (equal); writing – review and editing (lead).

## CONFLICT OF INTEREST

The authors declare no competing interests.

## Supporting information


**Appendix S1** Supporting Information

**Appendix S2** Supporting Information

## Data Availability

The raw sequencing data of the transcriptome and 16S rRNA generated in this study has been deposited in the NCBI SRA database (Bioproject ID: PRJNA858747 and PRJNA858286). The raw metabolome data after processing is shown in Supporting Information (Metabolite raw data.xlsx).
