# Supplementary material for: The efficacy of anti‐proteolytic peptide R7I in intestinal inflammation, function, microbiota, and metabolites by multi‐omics analysis in murine bacterial enteritis
Source: Bioeng Transl Med. 2022 Nov 8;8(2):e10446. doi: 10.1002/btm2.10446 (PMC10013768; doi:10.1002/btm2.10446)
Supplement: Supplementary file 2 — Appendix S2 Supporting Information [file BTM2-8-e10446-s002.docx]

**The efficacy of anti-proteolytic peptide R7I in intestinal inflammation, function, microbiota, and metabolites by multi-omics analysis in murine bacterial enteritis**

Taotao Sun, Xuesheng Liu, Yunzhe Su, Zihang Wang, Baojing Cheng, Na Dong, Jiajun Wang^**^, and Anshan Shan^*^

Laboratory of Molecular Nutrition and Immunity, the Institute of Animal Nutrition, Northeast Agricultural University, Harbin 150030, China.

**Serum, gastric juice, and small intestinal fluids without the interference of the experiment**

| **Items** | **1h** | **4h** | **8h** |
| --- | --- | --- | --- |
| GAJ + *E. coli* | inactive | inactive | inactive |
| SIJ + *E. coli* | inactive | inactive | inactive |
| Serum + *E. coli* | inactive | inactive | inactive |
| GAJ + PBS2.0 | sterile | sterile | sterile |
| SIJ + PBS7.0 | sterile | sterile | sterile |
| Serum + PBS7.0 | sterile | sterile | sterile |

GAJ, Gastric juice; SIJ, Small intestinal juice; PBS2.0 and PBS7.0, phosphate buffered saline (pH = 2.0 or pH = 7.0); *E. coli*, *Escherichia coli 25922*.

# Antibody

Internal reference β-actin (AA128) and horseradish peroxidase-labeled goat anti-rabbit IgG (A0208) from Shanghai Beyotime, China. Other antibodies from Affinity Biosciences, China (IL-4 Antibody, Art No. AF5142; IL-6 Art No. DF6087; IL-10 Antibody, Art No. DF6894; TNF alpha Antibody, Art No. DF6080).

# Metabolite testing parameters

Agilent 1290 InfinityⅡUHPLC system coupled to an Agilent 6545 UHD and Accurate-Mass Q-TOF/MS was used for LC-MS analysis. The chromatographic column used was Waters Waters XSelect ○R HSS T3 (2.5μm 100*2.1mm). Mobile phase: A: aqueous solution with 0.1% formic acid. B: acetonitrile solution with 0.1% formic acid. Flow rate: 0.4 ml/min. Column temperature: 40℃. Injection volume: 4 μl. Gradient elution condition optimized: 0-3min, 20% B;3-9min, 20-95% B;9-13min, 95% B;13-13.1min, 95-5% B;13.1-16min, 5% B. Mass spectrometry was operated in both positive and negative ion modes. The parameters optimized were as follows. Capillary voltage: 4.5 kV in positive mode and 3.5 kV in negative mode. Drying gas flow: 8 l/min in positive mode and 10 l/min in negative mode. Gas temperature: 325℃. Nebulizer pressur: 20 psig. Fragmentor voltage: 120 V. Skimmer voltage: 45 V. Mass range: m/z 50-1500.


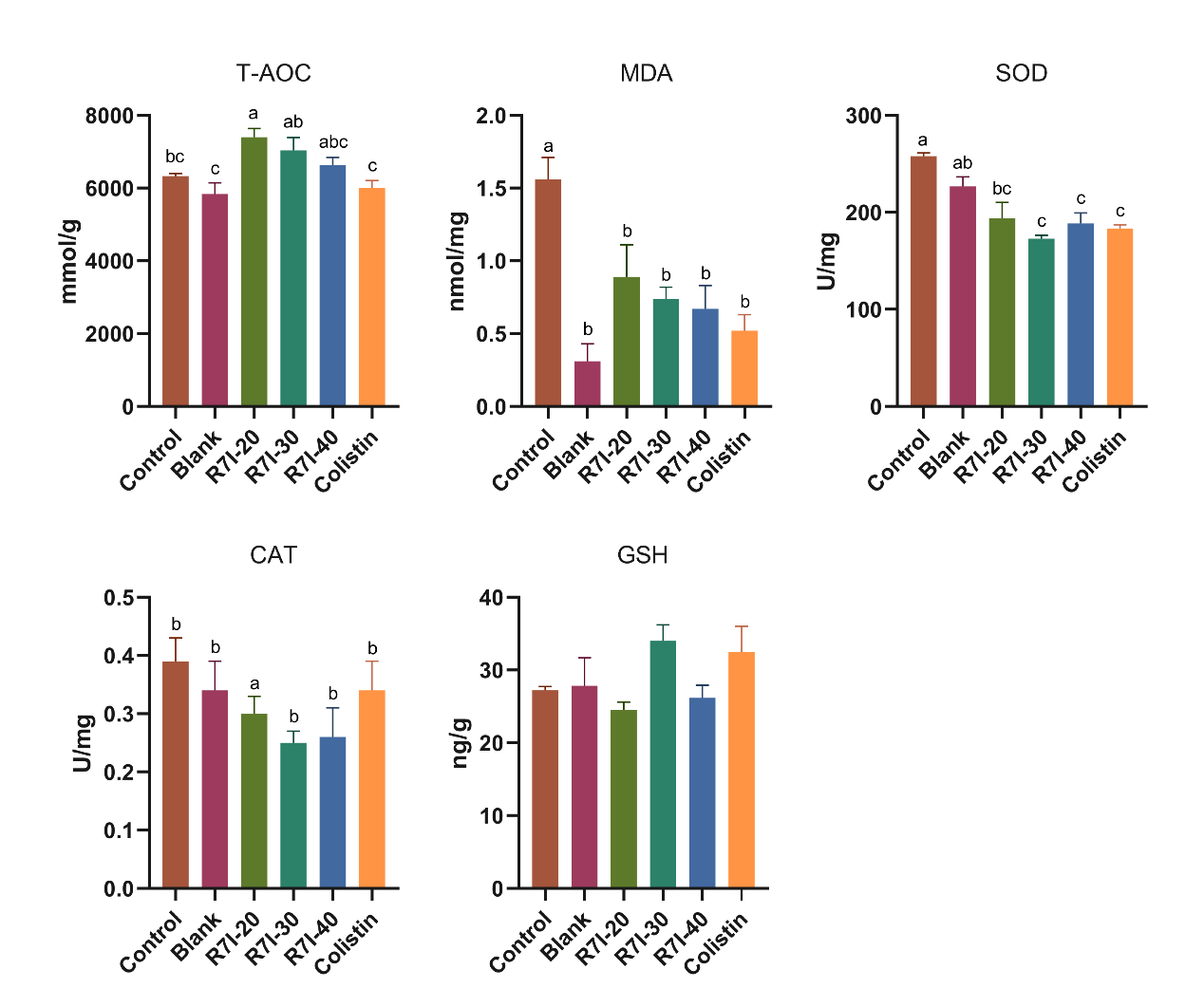


**Figure S1.** Antioxidant levels in the small intestine of mice. The data was presented as mean ± SEM (n = 6). One-way ANOVA with the Tukey post-test was used to determine statistical significance. A p-value of <0.05 was regarded as significant.

**Table S1** Design and synthesis of the mouse RT-PCR primers.

| Gene | Sequences (5'→3') | Fragments sizes | Gen Bank No. |
| --- | --- | --- | --- |
| IL-4 | F: GGTCTCAACCCCCAGCTAGT | 102 bp | NM_021283.2 |
|  | R: GCCGATGATCTCTCTCAAGTGAT |  |  |
| IL-6 | F: CTCCCAACAGACCTGTCTATAC | 97 bp | NM_031168.2 |
|  | R: CCATTGCACAACTCTTTTCTCA |  |  |
| IL-10 | F: TTCTTTCAAACAAAGGACCAGC | 81 bp | NM_010548.2 |
|  | R: GCAACCCAAGTAACCCTTAAAG |  |  |
| IL-1β | F: GAAATGCCACCTTTTGACAGTG | 116 bp | NM_008361.4 |
|  | R: TGGATGCTCTCATCAGGACAG |  |  |
| TNF-α | F: ATGTCTCAGCCTCTTCTCATTC | 179 bp | NM_013693.3 |
|  | R: GCTTGTCACTCGAATTTTGAGA |  |  |
| Occludin | F: TGCTTCATCGCTTCCTTAGTAA | 155 bp | NM_001360536.1 |
|  | R: GGGTTCACTCCCATTATGTACA |  |  |
| ZO-1 | F: CTGGTGAAGTCTCGGAAAAATG | 97 bp | NM_009386.2 |
|  | R: CATCTCTTGCTGCCAAACTATC |  |  |
| Claudin 1 | F: AGATACAGTGCAAAGTCTTCGA | 86 bp | NM_016674.4 |
|  | R: CAGGATGCCAATTACCATCAAG |  |  |
| β-actin | F: CTACCTCATGAAGATCCTGACC | 100 bp | NM_007393.5 |
|  | R: CACAGCTTCTCTTTGATGTCAC |  |  |

**Table S2** KEGG pathway enrichment of DEGs in the small intestine (Control vs. R7I-20).

| Pathway ID | Pathway name | Up-regulated genes | Down-regulated genes | P-value |
| --- | --- | --- | --- | --- |
| mmu04974 | Protein digestion and absorption | Slc7a9, Dpp4, Slc6a19, Slc16a10, Mep1b, Slc1a1, Xpnpep2, Ace2, Kcnk5, Slc7a7, Slc15a1, Atp1a1, Slc7a15, Mep1a, Atp1b1, Slc3a1. | Col1a1, Col1a2, Col5a2, Atp1a3, Prcp, Prss2, Cpa1. | 4.12 × 10^-9^ |
| mmu04975 | Fat digestion and absorption | Plpp2, Abcg8, Abcg5, Mogat2, Apob, Dgat2, Agpat2, Dgat1, Npc1l1, Pla2g12b, Mttp, Apoa1. | Pla2g2d | 1.47 × 10^-7^ |
| mmu04976 | Bile secretion | Slc22a1, Slc5a1, Slc51b, Abcg8, Abcg5, Slc51a, Slc4a4, Nr1h4, Atp1a1, Abcb1a, Atp1b1, Slc10a2, Abcg2, Hmgcr, Ldlr, Baat, Abcc2. | Atp1a3, Ephx1 | 1.57× 10^-7^ |
| mmu04978 | Mineral absorption | Slc26a6, Slc39a4, Slc5a1, Slc6a19, Slc26a3, Atp1a1, Clcn2, Slc31a1, Atp1b1, Heph, Slc30a1 | Atp1a3 | 2.22 × 10^-6^ |
| mmu04973 | Carbohydrate digestion and absorption | Slc5a1, Hkdc1, Mgam, Slc2a5, Atp1a1, Sis, Atp1b1, G6pc | Prkcb, Atp1a3, Akt3 | 3.16 × 10^-5^ |
| mmu04964 | Proximal tubule bicarbonate reclamation | Slc4a4, Atp1a1, Pck1, Atp1b1, Gls, Slc25a10 | Atp1a3 | 0.0002 |
| mmu03320 | PPAR signaling pathway | Acox2, Slc27a2, Slc27a4, Acsl5, Acaa1b, Pck1, Apoa1, Aqp7, Apoc3, Acox1, Gk, Cyp27a1 | Scd1, Fabp5 | 0.0005 |
| mmu04977 | Vitamin digestion and absorption | Lrat, Slc52a3, Tcn2, Cubn, Apob, Apoa1 | - | 0.0015 |
| mmu04979 | Cholesterol metabolism | Abcg8, Abcg5, Soat2, Pcsk9, Apob, Apoa1, Apoc3, Ldlr, Cyp27a1 | - | 0.0022 |
| mmu00100 | Steroid biosynthesis | Soat2, Lss, Hsd17b7, Fdft1, Sqle, Nsdhl, Dhcr24, Sc5d, Dhcr7 | - | 4.95 × 10^-7^ |
| mmu00830 | Retinol metabolism | Aldh1a7, Cyp3a25, Cyp3a13, Aldh1a1, Ugt2b34, Lrat, Cyp2c65, Ugt1a1, Dgat1, Cyp3a11, Rdh7, Rdh16, Cyp2b10, Rdh9, Cyp2c68, Ugt2b35, Ugt2b5, Cyp2c55, Bco1, Cyp2s1 | - | 5.05 × 10^-7^ |
| mmu00140 | Steroid hormone biosynthesis | Cyp3a25, Cyp3a13, Ugt2b34, Sult2b1, Hsd11b2, Cyp2c65, Hsd17b7, Ugt1a1, Cyp2d26, Srd5a2, Hsd3b3, Hsd3b2, Cyp3a11, Cyp2b10, Cyp2c68, Ugt2b35, Ugt2b5, Cyp2c55 | - | 4.81 × 10^-6^ |
| mmu00564 | Glycerophospholipid metabolism | Plpp2, Lpin3, Pcyt2, Cds1, Dgkd, Agpat2, Pla2g12b, Dgka, Gpd1, Cdipt, Pisd, Chka, Lpcat3, Etnppl | Pla2g2d | 0.0006 |
| mmu00561 | Glycerolipid metabolism | Plpp2, Lpin3, Mogat2, Akr1b7, Dgkd, Dgat2, Agpat2, Dgat1, Dgka, Glyctk, Gk | - | 0.0010 |
| mmu00591 | Linoleic acid metabolism | Cyp3a25, Cyp3a13, Cyp2c65, Pla2g12b, Cyp3a11, Cyp2j6, Cyp2c68, Cyp2c55 | Pla2g2d | 0.0025 |
| mmu04146 | Peroxisome | Acox2, Slc27a2, Acsl5, Acaa1b, Abcd3, Mvk, Abcd4, Acox1, Baat, Pecr, Ephx2, Idh1 | Far1 | 0.0017 |
| mmu04514 | Cell adhesion molecules (CAMs) | H2-Q1, Cldn15, H2-Q2, Nectin3, Cldn7, H2-T3, Cldn3, Itgb2l, H2-Bl, Gm8909, Ocln, Nectin2, Itga6, Ptprf, Neo1, Sdc1 | Glycam1, Vcam1, H2-DMb2, H2-Ob, H2-Oa, Siglec1, H2-Eb2, Sdc3, Cd22 | 9.33 × 10^-6^ |
| mmu02010 | ABC transporters | Abcg8, Abcg5, Abcb1a, Abcd3, Abcb10, Abcd4, Abcg2, Abcc6, Abcc2 | - | 0.0022 |
| mmu05204 | Chemical carcinogenesis | Cyp3a25, Cyp3a13, Ugt2b34, Gsta1, Cyp2c65, Mgst3, Ugt1a1, Mgst2, Cyp3a11, Cyp2b10, Cyp2c68, Ugt2b35, Ugt2b5, Cyp2c55 | Ephx1 | 0.0006 |

**Table S3** KEGG pathway enrichment of DEGs in the liver (Control vs. R7I-20).

| Pathway ID | Pathway name | Up-regulated genes | Down-regulated genes | P-value |
| --- | --- | --- | --- | --- |
| mmu00100 | Steroid biosynthesis | Sc5d, Lss, Msmo1, Cyp51 | - | 1.76 × 10^-6^ |
| mmu00071 | Fatty acid degradation | Cyp4a12b, Acat1, Hadha | - | 0.0019 |
| mmu00480 | Glutathione metabolism | Nat8, Nat8f2, Nat8f5 | - | 0.0039 |
| mmu00900 | Terpenoid backbone biosynthesis | Fdps, Acat1 | - | 0.0055 |
| mmu00650 | Butanoate metabolism | Acat1, Hadha | - | 0.0075 |
| mmu03320 | PPAR signaling pathway | Cyp4a12b, Fads2, Apoa5 | - | 0.0080 |
| mmu00640 | Propanoate metabolism | Acat1, Hadha | - | 0.0104 |
| mmu04152 | AMPK signaling pathway | Igf1, Adipor2, Fbp1 | - | 0.0220 |
| mmu00380 | Tryptophan metabolism | Acat1,Hadha | - | 0.0235 |
| mmu00280 | Valine, leucine and isoleucine degradation | Acat1,Hadha | - | 0.0301 |
| mmu00310 | Lysine degradation | Acat1, Hadha | - | 0.0321 |

**Table S4** KEGG pathway enrichment in the small intestine (Blank vs. Control).

| Pathway ID | Pathway | Up | Down | P-value |
| --- | --- | --- | --- | --- |
| mmu04974 | Protein digestion and absorption | 6 | 21 | 4.28 × 10^-6^ |
| mmu00564 | Glycerophospholipid metabolism | 2 | 22 | 0.0002 |
| mmu00480 | Glutathione metabolism | 5 | 13 | 0.0008 |
| mmu04975 | Fat digestion and absorption | 1 | 12 | 0.0008 |
| mmu04380 | Osteoclast differentiation | 19 | 7 | 0.0009 |
| mmu04978 | Mineral absorption | 2 | 11 | 0.0009 |
| mmu04610 | Complement and coagulation cascades | 19 | 2 | 0.0009 |
| mmu04976 | Bile secretion | 0 | 19 | 0.0017 |
| mmu04964 | Proximal tubule bicarbonate reclamation | 0 | 9 | 0.0017 |
| mmu02010 | ABC transporters | 0 | 14 | 0.0017 |
| mmu04514 | Cell adhesion molecules (CAMs) | 12 | 18 | 0.0018 |
| mmu04662 | B cell receptor signaling pathway | 16 | 1 | 0.0019 |
| mmu04640 | Hematopoietic cell lineage | 15 | 5 | 0.0040 |
| mmu00983 | Drug metabolism - other enzymes | 4 | 15 | 0.0044 |
| mmu05140 | Leishmaniasis | 13 | 3 | 0.0045 |
| mmu04973 | Carbohydrate digestion and absorption | 2 | 10 | 0.0045 |
| mmu00561 | Glycerolipid metabolism | 1 | 14 | 0.0045 |
| mmu04977 | Vitamin digestion and absorption | 1 | 7 | 0.0062 |
| mmu04972 | Pancreatic secretion | 7 | 14 | 0.0063 |
| mmu00100 | Steroid biosynthesis | 1 | 6 | 0.0118 |
| mmu00051 | Fructose and mannose metabolism | 2 | 8 | 0.0118 |

**Table S5** KEGG pathway enrichment in the small intestine (Blank vs. R7I-20).

| Pathway ID | Pathway | Up | Down | P-value |
| --- | --- | --- | --- | --- |
| mmu05140 | Leishmaniasis | 7 | 1 | 0.0003 |
| mmu04610 | Complement and coagulation cascades | 7 | 1 | 0.0015 |
| mmu04145 | Phagosome | 9 | 1 | 0.0049 |
| mmu00590 | Arachidonic acid metabolism | 2 | 5 | 0.0049 |
| mmu05152 | Tuberculosis | 10 | 0 | 0.0049 |
| mmu05144 | Malaria | 2 | 3 | 0.0210 |

**Table S6** KEGG pathway enrichment in the liver (Blank vs. Control).

| Pathway ID | Pathway | Up | Down | P-value |
| --- | --- | --- | --- | --- |
| mmu04380 | Osteoclast differentiation | 63 | 4 | 6.07 × 10^-10^ |
| mmu05132 | Salmonella infection | 41 | 6 | 1.31 × 10^-9^ |
| mmu04146 | Peroxisome | 9 | 41 | 1.18 × 10^-8^ |
| mmu05145 | Toxoplasmosis | 50 | 8 | 6.71 × 10^-8^ |
| mmu04668 | TNF signaling pathway | 49 | 6 | 6.83 × 10^-7^ |
| mmu05323 | Rheumatoid arthritis | 42 | 3 | 1.05 × 10^-6^ |
| mmu04621 | NOD-like receptor signaling pathway | 70 | 5 | 2.14 × 10^-6^ |
| mmu00830 | Retinol metabolism | 4 | 44 | 3.31 × 10^-6^ |
| mmu00140 | Steroid hormone biosynthesis | 7 | 39 | 5.38 × 10^-6^ |
| mmu01040 | Biosynthesis of unsaturated fatty acids | 5 | 18 | 8.04 × 10^-6^ |
| mmu00120 | Primary bile acid biosynthesis | 3 | 12 | 1.10 × 10^-6^ |
| mmu04062 | Chemokine signaling pathway | 71 | 8 | 1.12 × 10^-6^ |
| mmu05150 | Staphylococcus aureus infection | 27 | 5 | 1.15 × 10^-6^ |
| mmu04979 | Cholesterol metabolism | 7 | 20 | 0.0001 |
| mmu05321 | Inflammatory bowel disease (IBD) | 28 | 3 | 0.0001 |
| mmu04064 | NF-kappa B signaling pathway | 38 | 4 | 0.0002 |
| mmu04662 | B cell receptor signaling pathway | 30 | 4 | 0.0002 |
| mmu00010 | Glycolysis / Gluconeogenesis | 15 | 18 | 0.0002 |
| mmu00071 | Fatty acid degradation | 0 | 27 | 0.0002 |
| mmu04620 | Toll-like receptor signaling pathway | 40 | 4 | 0.0004 |
| mmu00590 | Arachidonic acid metabolism | 17 | 22 | 0.0005 |

**Table S7** KEGG pathway enrichment in the liver (Blank vs. R7I-20).

| Pathway ID | Pathway | Up | Down | P-value |
| --- | --- | --- | --- | --- |
| mmu04145 | Phagosome | 80 | 13 | 6.95 × 10^-12^ |
| mmu03010 | Ribosome | 69 | 3 | 4.61 × 10^-10^ |
| mmu05140 | Leishmaniasis | 41 | 2 | 2.49 × 10^-8^ |
| mmu04380 | Osteoclast differentiation | 59 | 6 | 2.49 × 10^-8^ |
| mmu04662 | B cell receptor signaling pathway | 31 | 7 | 1.60 × 10^-8^ |
| mmu00830 | Retinol metabolism | 3 | 44 | 3.03 × 10^-5^ |
| mmu04621 | NOD-like receptor signaling pathway | 65 | 8 | 4.11 × 10^-5^ |
| mmu05145 | Toxoplasmosis | 43 | 9 | 6.19 × 10^-5^ |
| mmu04668 | TNF signaling pathway | 44 | 7 | 7.45 × 10^-5^ |
| mmu05169 | Epstein-Barr virus infection | 71 | 19 | 9.74 × 10^-5^ |
| mmu04064 | NF-kappa B signaling pathway | 38 | 6 | 0.0001 |
| mmu05418 | Fluid shear stress and atherosclerosis | 45 | 17 | 0.0001 |

**Table S8** Positive differential metabolites (Control vs. R7I-20).

| HMDB ID | Name | Formula | VipV | Logfc | P-value |
| --- | --- | --- | --- | --- | --- |
| HMDB0012394 | PS (18:1(9Z)/20:4(5Z,8Z,11Z,14Z)) | C44H76NO10P | 2.58 | 1.34 | 0.0005 |
| HMDB0009778 | PI (16:0/16:0) | C41H79O13P | 2.57 | 1.3842 | 0.0005 |
| HMDB0114113 | PE-NMe2 (18:3 (6Z,9Z,12Z)/20:5 (5Z,8Z,11Z,14Z,17Z)) | C45H74NO8P | 2.51 | 1.32 | 0.0016 |
| HMDB0009144 | PE (18:3 (6Z,9Z,12Z)/22:6 (4Z,7Z,10Z,13Z,16Z,19Z)) | C45H72NO8P | 2.47 | 1.72 | 0.0026 |
| HMDB0008845 | PE (14:0/22:5 (4Z,7Z,10Z,13Z,16Z)) | C41H72NO8P | 2.33 | 1.00 | 0.0082 |
| HMDB0002596 | Deoxycholic acid 3-glucuronide | C30H48O10 | 2.28 | -1.53 | 0.0100 |
| HMDB0010404 | LysoPC(22:6 (4Z,7Z,10Z,13Z,16Z,19Z)/0:0) | C30H50NO7P | 2.25 | -1.50 | 0.0122 |
| HMDB0012252 | Linoleoyl ethanolamide | C20H37NO2 | 2.21 | -0.94 | 0.0159 |
| HMDB0071228 | TG (8:0/14:0/10:0) | C35H66O6 | 2.11 | -3.48 | 0.0200 |
| HMDB0113915 | PE-NMe2 (14:1 (9Z)/22:6 (4Z,7Z,10Z,13Z,16Z,19Z)) | C43H72NO8P | 2.02 | 1.46 | 0.0391 |
| HMDB0001469 | Undecaprenyl diphosphate | C55H92O7P2 | 1.98 | -1.05 | 0.0443 |

Filter: *P* < 0.05, VipV > 1, and | Logfc | > 1. HMDB, Human Metabolome Database; vipV, VIP value of OPLS-DA model; Logfc, Log value of fold change based on 2 for R7I-20/Control.

**Table S9** Negative differential metabolites (Control vs. R7I-20).

| HMDB ID | Name | Formula | VipV | Logfc | P-value |
| --- | --- | --- | --- | --- | --- |
| HMDB0000237 | Propionic acid | C3H6O2 | 2.41 | -1.66 | 0.0097 |
| HMDB0000202 | Methylmalonic acid | C4H6O4 | 2.37 | -2.18 | 0.0050 |
| HMDB0113059 | PE-NMe (16:0/24:0) | C46H92NO8P | 2.34 | 2.66 | 0.0122 |
| HMDB0000408 | 2-Methyl-3-ketovaleric acid | C6H10O3 | 2.31 | -1.11 | 0.0222 |
| HMDB0061660 | 2(R)-hydroxydocosanoic acid | C22H44O3 | 2.24 | 1.22 | 0.0307 |
| HMDB0072845 | MG (13:0/0:0/0:0) | C16H32O4 | 2.16 | -1.77 | 0.0348 |
| HMDB0060647 | 4-hydroxy ketorolac | C15H13NO4 | 2.14 | -1.65 | 0.0093 |
| HMDB0056386 | DG (22:6n3/0:0/22:6n3) | C47H68O5 | 2.10 | 1.46 | 0.0359 |
| HMDB0011494 | LysoPE (0:0/22:5 (4Z,7Z,10Z,13Z,16Z)) | C27H46NO7P | 2.08 | -1.06 | 0.0228 |
| HMDB0008194 | PC (18:3 (6Z,9Z,12Z)/P-18:1 (11Z)) | C44H80NO7P | 1.77 | -1.75 | 0.0444 |
| HMDB0031874 | N-gamma-Glutamyl-S-allylcysteine | C11H18N2O5S | 1.76 | 2.19 | 0.0479 |

Filter: *P* < 0.05, VipV > 1, and | Logfc | > 1. HMDB, Human Metabolome Database; vipV, VIP value of OPLS-DA model; Logfc, Log value of Fold Change based on 2 for group R7I-20/Control.

**Table S10** Negative differential metabolites (Blank vs. Control).

| HMDB ID | Name | Formula | VipV | Logfc | P-value |
| --- | --- | --- | --- | --- | --- |
| HMDB0062207 | 3alpha,7alpha,12alpha-trihydroxy-5beta-cholestanate | C27H46O5 | 1.45 | 5.28 | 0.038087 |
| HMDB0000063 | Cortisol | C21H30O5 | 1.81 | 4.75 | 0.001635 |
| HMDB0116660 | PG (i-12:0/i-12:0) | C30H59O10P | 1.89 | 4.65 | 4.94E-05 |
| HMDB0002496 | N-[(3a,5b,7a)-3-hydroxy-24-oxo-7-(sulfooxy)cholan-24-yl]-Glycine | C26H43NO8S | 1.74 | 4.61 | 0.00488 |
| HMDB0001952 | Hexacarboxylporphyrin III | C38H38N4O12 | 1.75 | 4.15 | 0.004713 |
| HMDB0006898 | Chenodeoxyglycocholic acid | C26H43NO5 | 1.43 | 3.82 | 0.039438 |
| HMDB0011494 | LysoPE (0:0/22:5 (4Z,7Z,10Z,13Z,16Z)) | C27H46NO7P | 1.88 | 3.63 | 0.000201 |
| HMDB0003752 | LysoPC (10:0/0:0) | C18H39NO7P | 1.49 | 3.58 | 0.029348 |
| HMDB0029779 | 2-O-Protocatechuoylalphitolic acid | C37H52O7 | 1.79 | 3.50 | 0.00135 |
| HMDB0000314 | 3b-Allotetrahydrocortisol | C21H34O5 | 1.79 | 3.45 | 0.001814 |
| HMDB0043684 | TG (15:0/18:4 (6Z,9Z,12Z,15Z)/O-18:0) | C54H98O5 | 1.81 | 3.41 | 0.000689 |
| HMDB0011686 | p-Cresol glucuronide | C13H16O7 | 1.57 | 3.39 | 0.020432 |
| HMDB0029315 | Asparagoside B | C33H56O9 | 1.70 | 3.34 | 0.004866 |
| HMDB0011499 | LysoPE (0:0/24:6 (6Z,9Z,12Z,15Z,18Z,21Z)) | C29H48NO7P | 1.53 | 3.27 | 0.025349 |
| HMDB0130621 | {[2,4,6-trihydroxy-3-methyl-5-(3-phenylpropanoyl)phenyl]methoxy}sulfonic acid | C17H18O8S | 1.64 | 3.23 | 0.009345 |
| HMDB0002579 | Glycochenodeoxycholic acid 3-glucuronide | C32H51NO11 | 1.92 | 3.15 | 1.37E-05 |
| HMDB0010331 | Palmitoyl glucuronide | C22H42O7 | 1.89 | 2.98 | 4.50E-05 |
| HMDB0038242 | Ascorbyl stearate | C24H42O7 | 1.81 | 2.89 | 0.001033 |
| HMDB0039942 | Notoginsenoside L | C53H90O22 | 1.63 | 2.72 | 0.013917 |
| HMDB0000518 | Chenodeoxycholic acid | C24H40O4 | 1.49 | -5.29 | 0.025474 |
| HMDB0115488 | PA (8:0/17:0) | C28H55O8P | 1.49 | -5.53 | 0.021603 |
| HMDB0114774 | PA (10:0/17:0) | C30H59O8P | 1.73 | -4.86 | 0.034385 |

Filter: *P* < 0.05, VipV > 1, and | Logfc | > 1. HMDB, Human Metabolome Database; vipV, VIP value of OPLS-DA model; Logfc, Log value of Fold Change based on 2 for group Control/Blank.

**Table S11** Positive differential metabolites (Blank vs. Control).

| HMDB ID | Name | Formula | VipV | Logfc | P-value |
| --- | --- | --- | --- | --- | --- |
| HMDB0006210 | Heptadecanoyl carnitine | C24H47NO4 | 1.75 | 7.61 | 0.0003 |
| HMDB0013326 | trans-2-Dodecenoylcarnitine | C19H35NO4 | 1.74 | 4.21 | 0.0003 |
| HMDB0034079 | Homodolichosterone | C29H48O5 | 1.73 | 2.86 | 0.0006 |
| HMDB0006460 | Arachidyl carnitine | C27H53NO4 | 1.72 | 2.36 | 0.0006 |
| HMDB0116817 | CL (8:0/8:0/8:0/18:2 (9Z,11Z)) | C51H94O17P2 | 1.72 | 9.02 | 0.0009 |
| HMDB0012937 | Dynorphin B (6-9) | C26H43N11O6 | 1.67 | 4.67 | 0.0017 |
| HMDB0000761 | Lithocholic acid | C24H40O3 | 1.66 | 4.65 | 0.0017 |
| HMDB0005773 | Endomorphin-1 | C34H38N6O5 | 1.65 | 3.76 | 0.0024 |
| HMDB0035607 | Saponin H | C36H58O10 | 1.61 | 2.08 | 0.0043 |
| HMDB0094685 | Hexadecyl Benzoic acid | C23H38O2 | 1.58 | 1.96 | 0.0053 |
| HMDB0000848 | Stearoylcarnitine | C25H49NO4 | 1.58 | 1.57 | 0.0054 |
| HMDB0060129 | 24-Oxo-1alpha,23,25-trihydroxyvitamin D3 | C27H42O5 | 1.56 | 2.32 | 0.0075 |
| HMDB0011687 | Phenylbutyrylglutamine | C15H20N2O4 | 1.44 | 2.78 | 0.0217 |
| HMDB0060359 | 2,3-Epoxymenaquinone | C21H24O3 | 1.44 | 2.45 | 0.0193 |
| HMDB0055529 | TG (18:4 (6Z,9Z,12Z,15Z)/22:6 (4Z,7Z,10Z,13Z,16Z,19Z)/22:6 (4Z,7Z,10Z,13Z,16Z,19Z)) | C65H94O6 | 1.40 | 3.28 | 0.0310 |
| HMDB0001198 | Leukotriene C4 | C30H47N3O9S | 1.40 | 1.98 | 0.0254 |
| HMDB0055133 | TG (22:5 (4Z,7Z,10Z,13Z,16Z)/22:6 (4Z,7Z,10Z,13Z,16Z,19Z)/22:5 (4Z,7Z,10Z,13Z,16Z)) | C69H102O6 | 1.40 | 2.43 | 0.0342 |
| HMDB0011538 | MG (0:0/18:2 (9Z,12Z)/0:0) | C21H38O4 | 1.40 | 1.86 | 0.0251 |
| HMDB0010561 | TG (22:6 (4Z,7Z,10Z,13Z,16Z,19Z)/22:5 (7Z,10Z,13Z,16Z,19Z)/22:6 (4Z,7Z,10Z,13Z,16Z,19Z)) | C69H100O6 | 1.40 | 2.39 | 0.0340 |
| HMDB0114113 | PE-NMe2 (18:3(6Z,9Z,12Z)/20:5 (5Z,8Z,11Z,14Z,17Z)) | C45H74NO8P | 1.76 | -1.56 | 1.7589 |
| HMDB0009151 | PE (18:3(9Z,12Z,15Z)/14:0) | C37H68NO8P | 1.72 | -1.13 | 1.7151 |
| HMDB0008845 | PE (14:0/22:5 (4Z,7Z,10Z,13Z,16Z)) | C41H72NO8P | 1.70 | -1.00 | 1.6980 |
| HMDB0115093 | PA (20:1(11Z)/15:0) | C38H73O8P | 1.69 | -1.72 | 1.6864 |

Filter: *P* < 0.05, VipV > 1, and | Logfc | > 1. HMDB, Human Metabolome Database; vipV, VIP value of OPLS-DA model; Logfc, Log value of Fold Change based on 2 for group Control/Blank.

**Table S12** Negative differential metabolites (Blank vs. R7I-20).

| HMDB ID | Name | Formula | VipV | Logfc | P-value |
| --- | --- | --- | --- | --- | --- |
| HMDB0000063 | Cortisol | C21H30O5 | 2.01 | 4.72 | 3.23E-05 |
| HMDB0116660 | PG(i-12:0/i-12:0) | C30H59O10P | 2.01 | 4.44 | 3.52E-06 |
| HMDB0033662 | (S)-5'-Deoxy-5'-(methylsulfinyl)adenosine | C11H15N5O4S | 2.00 | 2.80 | 3.92E-05 |
| HMDB0010355 | Cholestane-3,7,12,25-tetrol-3-glucuronide | C33H56O10 | 1.99 | 4.78 | 0.000125 |
| HMDB0010331 | Palmitoyl glucuronide | C22H42O7 | 1.98 | 3.21 | 7.57E-05 |
| HMDB0000314 | 3b-Allotetrahydrocortisol | C21H34O5 | 1.98 | 3.46 | 0.0001 |
| HMDB0029779 | 2-O-Protocatechuoylalphitolic acid | C37H52O7 | 1.94 | 2.93 | 0.0004 |
| HMDB0011494 | LysoPE (0:0/22:5 (4Z,7Z,10Z,13Z,16Z)) | C27H46NO7P | 1.93 | 2.55 | 0.0005 |
| HMDB0038242 | Ascorbyl stearate | C24H42O7 | 1.90 | 2.61 | 0.0007 |
| HMDB0010006 | PIP (20:3 (5Z,8Z,11Z)/18:0) | C50H92O18P2 | 1.86 | 1.97 | 0.0041 |
| HMDB0133010 | {[1-(4-methoxyphenyl)pentan-3-yl]oxy}sulfonic acid | C12H18O5S | 1.85 | 3.01 | 0.0016 |
| HMDB0002579 | Glycochenodeoxycholic acid 3-glucuronide | C32H51NO11 | 1.83 | 2.97 | 0.0021 |
| HMDB0030535 | Notoginsenoside E | C48H82O20 | 1.82 | 3.89 | 0.0038 |
| HMDB0116512 | PGP (a-13:0/a-13:0) | C32H64O13P2 | 1.82 | 2.34 | 0.0033 |
| HMDB0029315 | Asparagoside B | C33H56O9 | 1.81 | 3.80 | 0.0037 |
| HMDB0031874 | N-gamma-Glutamyl-S-allylcysteine | C11H18N2O5S | 1.78 | 2.84 | 0.0046 |
| HMDB0130621 | {[2,4,6-trihydroxy-3-methyl-5-(3-phenylpropanoyl)phenyl]methoxy}sulfonic acid | C17H18O8S | 1.77 | 3.52 | 0.0055 |
| HMDB0032521 | Stearyl citrate | C24H44O7 | 1.77 | 2.35 | 0.0057 |
| HMDB0013058 | S-(9-deoxy-delta9,12-PGD2)-glutathione | C30H47N3O10S | 1.74 | 1.27 | 0.0054 |
| HMDB0118991 | CL (8:0/12:0/18:2 (9Z,11Z)/18:2 (9Z,11Z)) | C65H118O17P2 | 1.73 | 2.76 | 0.0136 |
| HMDB0114774 | PA (10:0/17:0) | C30H59O8P | 1.94 | 4.41 | 0.0005 |
| HMDB0007937 | PC (15:0/18:0) | C41H82NO8P | 1.93 | 1.30 | 0.0009 |
| HMDB0037840 | N-(1-Deoxy-1-fructosyl)leucine | C12H23NO7 | 1.86 | 2.07 | 0.0047 |
| HMDB0135493 | 6-[5-(2-{[2-(3,4-dimethoxyphenyl)ethyl]-C-hydroxycarbonimidoyl}eth-1-en-1-yl)-2-methoxyphenoxy]-3,4,5-trihydroxyoxane-2-carboxylic acid | C26H31NO11 | 1.85 | 2.80 | 0.0026 |
| HMDB0039032 | trans-Grandmarin isovalerate | C20H24O7 | 1.81 | 1.41 | 0.0050 |

Filter: *P* < 0.05, VipV > 1, and | Logfc | > 1. HMDB, Human Metabolome Database; vipV, VIP value of OPLS-DA model; Logfc, Log value of Fold Change based on 2 for group R7I-20/Blank.

**Table S13** Positive differential metabolites (Blank vs. R7I-20).

| HMDB ID | Name | Formula | VipV | Logfc | P-value |
| --- | --- | --- | --- | --- | --- |
| HMDB0116817 | CL (8:0/8:0/8:0/18:2(9Z,11Z)) | C51H94O17P2 | 1.81 | 7.76 | 0.0058 |
| HMDB0006210 | Heptadecanoyl carnitine | C24H47NO4 | 1.96 | 6.91 | 0.0004 |
| HMDB0012937 | Dynorphin B (6-9) | C26H43N11O6 | 1.92 | 4.60 | 0.0010 |
| HMDB0000761 | Lithocholic acid | C24H40O3 | 1.91 | 4.58 | 0.0010 |
| HMDB0000112 | gamma-Aminobutyric acid | C4H9NO2 | 1.98 | 3.68 | 0.0003 |
| HMDB0001319 | Pyridoxine 5'-phosphate | C8H12NO6P | 1.80 | 3.50 | 0.0076 |
| HMDB0013078 | Stearoylethanolamide | C20H41NO2 | 1.88 | 3.15 | 0.0019 |
| HMDB0013326 | trans-2-Dodecenoylcarnitine | C19H35NO4 | 1.85 | 3.06 | 0.0029 |
| HMDB0000559 | 3-Methoxy-4-hydroxyphenylethyleneglycol sulfate | C9H12O7S | 1.87 | 2.95 | 0.0034 |
| HMDB0055529 | TG (18:4 (6Z,9Z,12Z,15Z)/22:6 (4Z,7Z,10Z,13Z,16Z,19Z)/22:6 (4Z,7Z,10Z,13Z,16Z,19Z)) | C65H94O6 | 1.62 | 2.80 | 0.0263 |
| HMDB0056288 | DG (18:2n6/0:0/22:4n6) | C44H74O5 | 1.51 | 2.72 | 0.0446 |
| HMDB0011492 | LysoPE (0:0/22:2(13Z,16Z)) | C27H52NO7P | 1.92 | 2.66 | 0.0010 |
| HMDB0112959 | PE-Nme (14:0/18:0) | C38H76NO8P | 1.51 | 2.66 | 0.0443 |
| HMDB0061700 | 2-Linoleoylglycerophosphocholine | C27H53NO7P | 1.89 | 2.59 | 0.0015 |
| HMDB0128185 | 4-hydroxy-5-(2,4,5-trihydroxyphenyl)pentanoic acid | C11H14O6 | 1.78 | 2.54 | 0.0086 |
| HMDB0001570 | Thymidine 3',5'-cyclic monophosphate | C10H13N2O7P | 1.69 | 2.52 | 0.0160 |
| HMDB0002006 | 2,3-Diaminopropionic acid | C3H8N2O2 | 1.75 | 2.44 | 0.0111 |
| HMDB0011538 | MG (0:0/18:2 (9Z,12Z)/0:0) | C21H38O4 | 1.72 | 2.36 | 0.0082 |
| HMDB0011533 | MG (0:0/16:0/0:0) | C19H38O4 | 1.76 | 2.32 | 0.0057 |
| HMDB0003550 | Calcidiol | C27H44O2 | 1.87 | 2.31 | 0.0027 |
| HMDB0000244 | Riboflavin | C17H20N4O6 | 1.50 | -3.07 | 0.0435 |
| HMDB0000879 | Tetrahydrodeoxycorticosterone | C21H34O3 | 1.52 | -2.53 | 0.0430 |
| HMDB0029353 | Galactosyl 4-hydroxyproline | C11H19NO8 | 1.54 | -2.00 | 0.0342 |

Filter: *P* < 0.05, VipV > 1, and | Logfc | > 1. HMDB, Human Metabolome Database; vipV, VIP value of OPLS-DA model; Logfc, Log value of Fold Change based on 2 for group R7I-20/Blank.
